# Orpinolide disrupts a leukemic dependency on cholesterol transport by inhibiting OSBP

**DOI:** 10.1038/s41589-024-01614-4

**Published:** 2024-06-21

**Authors:** Marko Cigler, Hana Imrichova, Fabian Frommelt, Lucie Caramelle, Laura Depta, Andrea Rukavina, Chrysanthi Kagiou, J. Thomas Hannich, Cristina Mayor-Ruiz, Giulio Superti-Furga, Sonja Sievers, Alison Forrester, Luca Laraia, Herbert Waldmann, Georg E. Winter

**Affiliations:** 1 CeMM Research Center for Molecular Medicine of the Austrian Academy of Sciences, Vienna, Austria; 2 Unit of Research of Biochemistry and Cell Biology (URBC), Namur Research Institute for Life Sciences (NARILIS), University of Namur, Namur, Belgium; 3 Department of Chemistry, Technical University of Denmark, Lyngby, Denmark; 4 Center for Physiology and Pharmacology, Medical University of Vienna, Vienna, Austria; 5 Department of Chemical Biology, Max-Planck Institute of Molecular Physiology, Dortmund, Germany; 6 Present Address: IRB Barcelona—Institute for Research in Biomedicine, The Barcelona Institute of Science and Technology, Barcelona, Spain

**Keywords:** Target identification, Chemical genetics, Cancer therapy, Membrane trafficking, Lipids

## Abstract

Metabolic alterations in cancer precipitate in associated dependencies that can be therapeutically exploited. To meet this goal, natural product-inspired small molecules can provide a resource of invaluable chemotypes. Here, we identify orpinolide, a synthetic withanolide analog with pronounced antileukemic properties, via orthogonal chemical screening. Through multiomics profiling and genome-scale CRISPR–Cas9 screens, we identify that orpinolide disrupts Golgi homeostasis via a mechanism that requires active phosphatidylinositol 4-phosphate signaling at the endoplasmic reticulum–Golgi membrane interface. Thermal proteome profiling and genetic validation studies reveal the oxysterol-binding protein OSBP as the direct and phenotypically relevant target of orpinolide. Collectively, these data reaffirm sterol transport as a therapeutically actionable dependency in leukemia and motivate ensuing translational investigation via the probe-like compound orpinolide.

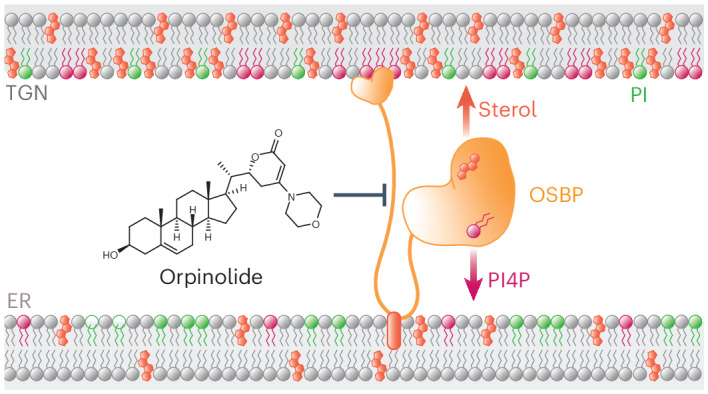

## Abstract (web summary)

Chemical, genetic and proteomic approaches were used to elucidate the mode of action of orpinolide, a withanolide-inspired OSBP inhibitor, revealing sterol transport as a druggable metabolic dependency in leukemia.

## Main

Rewiring of metabolic networks contributes to the development, progression and resistance acquisition of cancers, including leukemia, and is a general hallmark of cancer^1,2^. Individuals with acute myeloid leukemia (AML) or acute lymphoblastic leukemia (ALL) display several metabolic changes, including alterations in lipid and cholesterol metabolism to enable rapid proliferation, thus establishing motivation to exploit associated metabolic dependencies^3,4^. A suite of genome-scale genetic perturbation screens, for instance, CRISPR–Cas9 knockout screens, provide a rich catalog of cell-autonomous essential cancer cell functions, including dependencies on lipid or sterol metabolic pathways. However, redundancy and genetic buffering inherent to biological systems often require modulation of more than one protein or modulation that goes beyond functional inhibition. These characteristics are often outside the reach of genetic perturbations but are attainable via small molecules.

Natural products (NPs) are an invaluable source of bioactive molecules with remarkable therapeutic potential. Approximately one-third of all US Food and Drug Administration (FDA)-approved drugs in the last three decades originated from NPs or their derivatives^5^. The intricate biological relevance of NPs is defined by the rich and diverse chemical space embedded in their structures^6^. Yet, this structural complexity often encumbers the target identification and optimization of NP-derived bioactive compounds. To overcome these limitations, new strategies for identification and synthesis of simplified NP scaffolds are required.

The expansion of tangible chemical space can on one hand be achieved through biology-oriented synthesis (BIOS)^7^. BIOS simplifies NPs into core scaffolds with retained biological activities. These scaffolds in turn serve as starting points for the synthesis and further diversification of focused NP-inspired compound collections. On the other hand, fragmentation of NPs and their de novo combination allows the design of naturally nonoccurring scaffolds, so-called pseudo-NPs^8^. In both cases, fine-tuning of chemical space through simplification of complex parental NPs can yield compounds with improved biological activities, making them attractive starting points for drug discovery.

Withanolides, a highly abundant class of plant-derived polyoxygenated steroidal lactones, have been associated with diverse biological activities since the discovery and isolation of archetypal withaferin A^9^. Notably, withaferin A and other derivatives possess potent anti-inflammatory and antitumor effects by modulating pathways such as NF-κB, MAPK and WNT or inhibiting the ubiquitin–proteasome system^10–13^. It is thus not surprising that the withanolide scaffold has inspired the design of focused compound collections, prompting the identification of inhibitors of Hedgehog or Wnt signaling^14,15^. Of note, withanolide D was shown to induce apoptosis in leukemia cells through modulating ceramide production, while statin-mediated inhibition of the mevalonate pathway has recently been shown to be efficacious in early T cell progenitor ALL^16,17^. Collectively, these findings establish a rationale to broadly assay the efficacy of withanolide-inspired small molecules in AML and ALL cellular models.

Here, we couple leukemia cell line viability screens with morphological profiling of a focused withanolide-inspired compound collection and identify structurally similar derivatives with pronounced antiproliferative effects. This provided motivation to conduct unbiased, multiomics target identification studies for the lead compound orpinolide (W7). Integrating quantitative proteomics and transcriptomics was indicative of functional impairment of Golgi homeostasis and cholesterol biosynthesis. To map cellular effectors of the antiproliferative consequences of orpinolide, we conducted a genome-scale CRISPR–Cas9 screen that revealed a requirement of active phosphatidylinositol 4-phosphate (PtdIns4P, also known as PI4P) signaling at endoplasmic reticulum (ER)–Golgi membrane contact sites (MCSs). Using thermal proteome profiling (TPP), we ultimately uncover oxysterol-binding protein (OSBP) and its close ortholog ORP4 as direct targets of orpinolide. Importantly, we verify that OSBP is the predominant metabolic dependency in leukemia cells and that ORP4 is largely dispensable, and we reaffirm sterol transport at ER–Golgi MCSs as a potentially druggable vulnerability in leukemia cells.

## Results

### Phenotypic profiling of withanolide-inspired compounds

**Extended Data Fig. 1:**
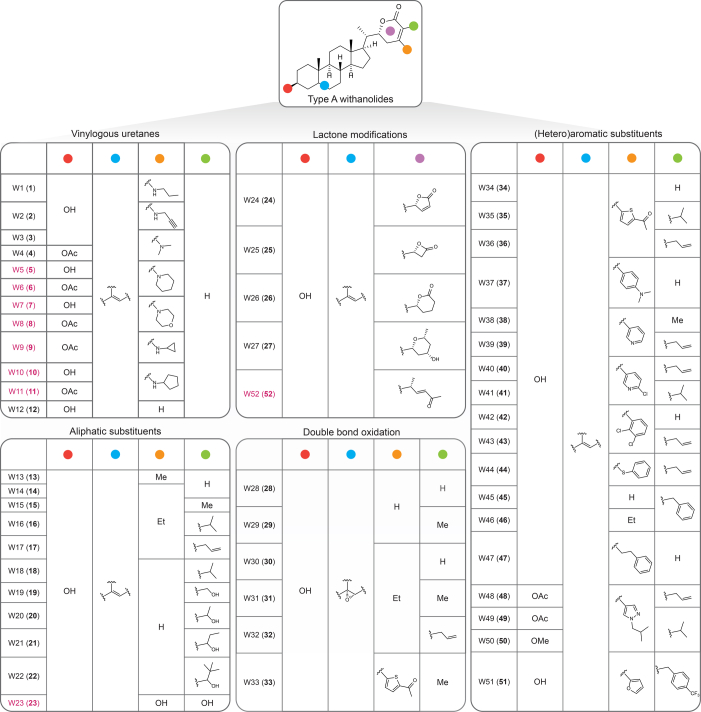
Withanolide-inspired compound collection. Structural overview of the 52-membered compound library based on the type A withanolide scaffold. The cytotoxic vinylogous uretanes W5-W11, their putative hydrolysis product W23 and the ring-fragmented product W52 are highlighted as well. SMILES representations of the compound collection are available in the Supplementary Table 1.

To systematically probe the effects of withanolides on leukemia cells, we selected a previously described compound collection based on the core steroid scaffold of type A withanolides (Extended Data Fig. 1 and Supplementary Table 1)^15^. Generated by BIOS, the structural diversity within this focused library was predominantly introduced by derivatizing the δ-lactone side chain while keeping the core sterol skeleton mostly unmodified. Previously studied in the context of cell signaling pathway modulators, several derivatives were shown to inhibit Wnt/β-catenin signaling in cell-based assays^15^.

**Fig. 1:**
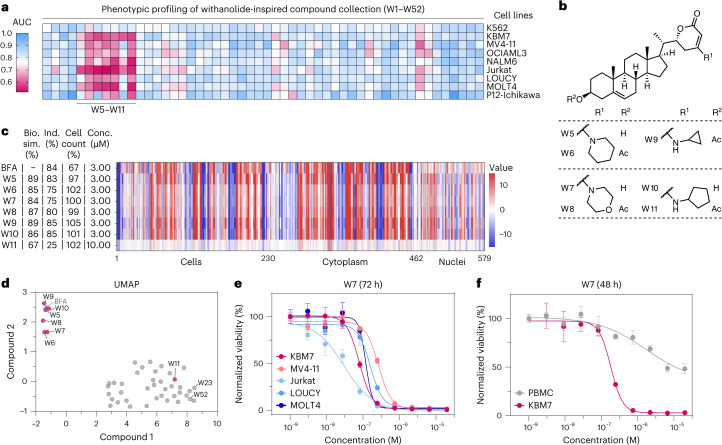
Orthogonal chemical profiling of a withanolide-inspired compound collection identifies W7. **a**, Heat map depicting results of the luminescence-based cell viability screening of a 52-membered withanolide-inspired compound collection in a panel of leukemia cells treated for 72 h. Results are represented as relative area under the curve (AUC) calculated from eight-concentration-point dose–response curves. See also Supplementary Table 1. **b**, Chemical structures of seven derivatives (W5–W11) with pronounced cytotoxicity in most of the tested cell lines. **c**, Cell painting profiles of BFA and withanolides W5–W11 over 579 cellular features. Biosimilarity (Bio. sim.) values represent the similarity of phenotypic profiles, whereas induction (Ind.) values represent the fraction of significantly changed cellular features. **d**, UMAP analysis of the cell painting assay data highlighting the biological similarity of withanolides W5–W10 (3 μM) to BFA (3 μM) over other withanolides (3 or 10 μM). The bioactivity fingerprint of the cytotoxic analog W11 (10 μM) differs from BFA. **e**, Dose-resolved normalized viability of the indicated leukemia cell lines after 72 h of W7 treatment (EC_50_ (KBM7) = 79.7 nM; EC_50_ (MV4-11) = 265.3 nM; EC_50_ (Jurkat) = 30.7 nM; EC_50_ (LOUCY) = 158.5 nM; EC_50_ (MOLT4) = 119.5 nM). Data are mean ± s.e.m.; *n* = 3 independent treatments. **f**, Dose-resolved normalized viability after 48 h of treatment of blood-isolated PBMCs or KBM7 cells with W7 (EC_50_ (KBM7) = 182.7 nM). Data are mean ± s.e.m.; *n* = 3 independent treatments. Source data

**Extended Data Fig. 2:**
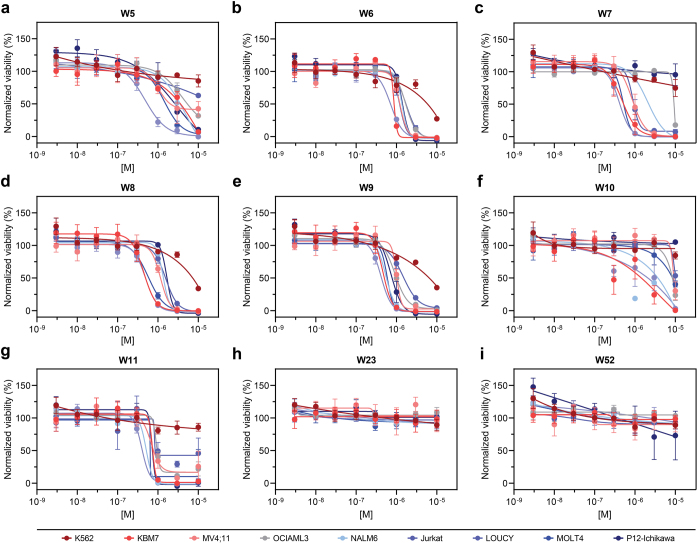
Phenotypic profiling of the withanolide-inspired compound collection in leukemia cells. (**a**-**g**) Representative dose response curves for seven cytotoxic vinylogous urethans (W5-W11) as well as their (**h**) putative hydrolysis product (W23) and (**i**) the ring-fragmented product (W52). Mean ± s.e.m.; n = 3 independent treatments. Source data

The 52-membered compound collection (W1–W52 (**1**–**52**)) was profiled in a cell line panel consisting of main lineages of leukemia, including chronic myeloid leukemia (K562 and KBM7), AML (MV4-11 and OCIAML3) and pre-B cell (NALM6) and T cell ALL (Jurkat, LOUCY, MOLT4 and P12-Ichikawa). Following a 72-h treatment at eight drug concentration points, we identified a cluster of compounds (W5–W11) that showed a pronounced cytotoxicity effect across all tested cell lines with the exception of K562 cells (Fig. 1a, Extended Data Fig. 2a–g and Supplementary Table 1). Intriguingly, W5–W11 are all vinylogous urethane analogs derived from aliphatic (hetero)cyclic primary or secondary amines (Fig. 1b), suggesting that the observed viability phenotype in leukemia requires certain structural/chemical features. This is further supported by the notion that, for example, the putative hydrolysis product of W5–W11 (W23) and the ring-fragmented product (W52) were not toxic to the tested cell lines (Extended Data Fig. 2h,i).

**Extended Data Fig. 3:**
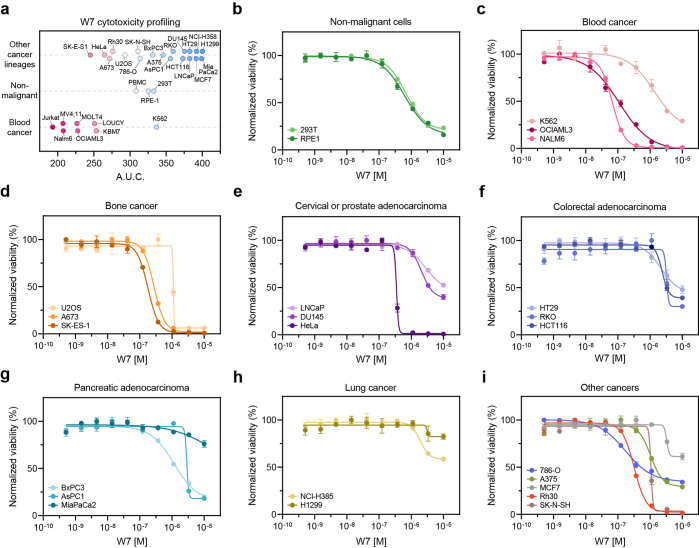
Cell viability profiling of W7 in additional non-malignant and non-leukemic cell lines. (**a**) Overview of the luminescence-based cell viability screening results for W7 across a panel of 30 different cell lines. The results are represented as area under the curve (A.U.C.), calculated from 10-concentration-point dose-response curves. The cells were exposed to the drug for 72 hours, with the exception of PBMCs, which had a 48-hour treatment period. (**b**-**i**) Dose-resolved, normalized viability of non-malignant (**b**), blood cancer (**c**), bone cancer (**d**), cervical/prostate adenocarcinoma (**e**), colorectal adenocarcinoma (**f**), pancreatic adenocarcinoma (**g**), lung cancer (**h**) and other cancer cells (**i**) after 72 h of W7 treatment. Mean ± s.e.m.; n = 3 independent treatments. Source data

Next, we used a cell painting assay^18^ to characterize morphological phenotypes induced by W5–W11. To contextualize the obtained morphological profiles, we compared the bioactivity fingerprints of the tested withanolides to a set of reference compounds with known mode of action and looked for possible biological similarities between them. To our surprise, six of seven cytotoxic analogs (W5–W10) showed a high similarity (>80%) to the fungal metabolite brefeldin A (BFA)^19,20^ (Fig. 1c). Notably, the morphological similarity was confined to the subset of withanolides that showed strong antileukemic properties (with the exception of W11), as visible in the uniform manifold approximation and projection (UMAP) analysis of recorded fingerprints (Fig. 1d). Although the similarity to BFA does not indicate that cytotoxic withanolides share the same targets, it is indicative of an involvement of the Golgi in the antileukemic properties of W5–W10. To further elucidate this connection, we prioritized W7 and assessed its cellular efficacy in dose-ranging validation experiments (Fig. 1e and Extended Data Fig. 3). W7 was particularly toxic in KBM7 cells and T cell leukemia-derived cells with half-maximal effective concentration (EC_50_) values in the lower nanomolar range. To ascertain whether this effect is exclusive to leukemia, we further assessed the efficacy of W7 in over 20 additional cell lines, encompassing both nonmalignant cells and various other cancer cell types. Indeed, we observed a clear hypersensitivity of leukemia cells to W7 exposure. However, the viabilities of selected nonleukemic cancer cell lines (including HeLa cells or the Ewing sarcoma cell lines SK-ES-1 and A673) were also affected by W7 (Extended Data Fig. 3d,e). Importantly, W7 did not substantially affect the viability of nonmalignant peripheral blood mononuclear cells (PBMCs), 293T cells or human retinal pigment epithelial RPE-1 cells, thus highlighting a potential therapeutic window (Fig. 1f and Extended Data Fig. 3b). Of note, unlike other vinylogous urethane withanolides, W7 did not show activity in cell-based Wnt pathway inhibition assays^15^.

### W7 disrupts Golgi homeostasis and cholesterol biosynthesis

**Fig. 2:**
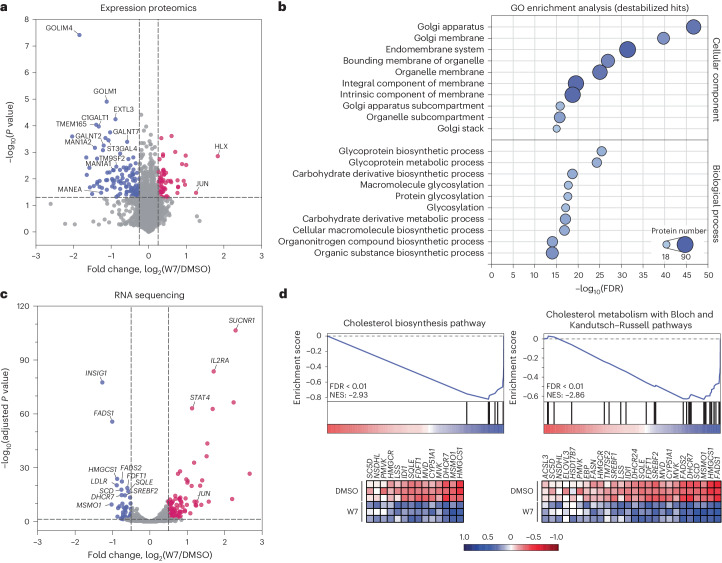
W7 treatment destabilizes Golgi proteins and represses cholesterol biosynthesis. **a**, Proteome-wide changes after 8 h of treatment of KBM7 cells with W7 (1 μM). The data are shown as log_2_-transformed fold change and −log_10_-transformed Benjamini–Hochberg-corrected one-way analysis of variance (ANOVA) *P* values compared to DMSO treatment; *n* = 2 biological replicates. See also Supplementary Table 2. **b**, Gene Ontology (GO) enrichment analysis of destabilized expression proteomic hits (highlighted in blue in **a**) for GO cellular components and GO biological processes. False discovery rates (FDRs; –log_10_-transformed) against the top ten enriched terms sorted by adjusted *P* value are shown. **c**, Changes in gene expression after 6 h of treatment of KBM7 cells with W7 (485 nM). The data are shown as log_2_-transformed fold change and −log_10_-transformed Benjamini–Hochberg-adjusted one-way ANOVA *P* values compared to DMSO treatment; *n* = 3 biological replicates. See also Supplementary Table 3. **d**, Gene set enrichment analysis (GSEA) of the top two WikiPathway gene sets from MSigDB significantly enriched in the differential gene expression profile of W7 against DMSO. Normalized enrichment scores (NES) and FDR are shown. Variance stabilizing transformation (VST)-normalized median-centered gene expression values of the leading edge subset of genes are depicted in the corresponding heat maps. Genes are ordered based on the metascore. Source data

**Extended Data Fig. 4:**
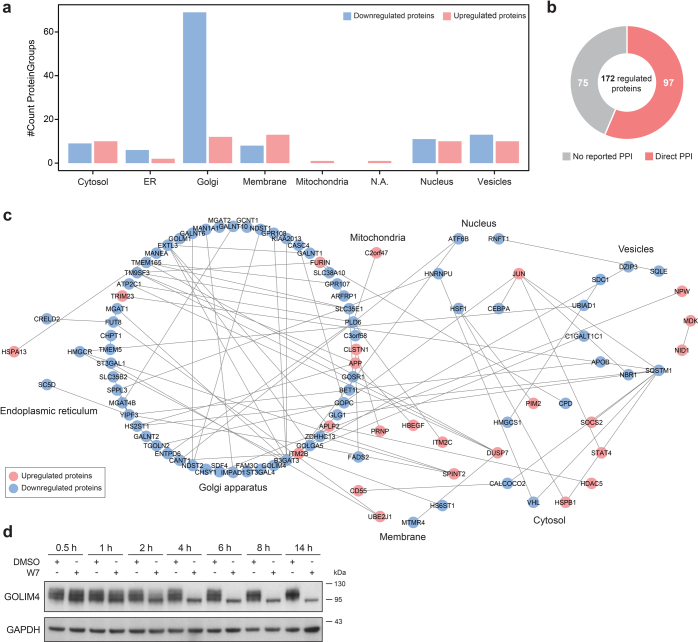
W7 induces global destabilization of Golgi-related proteins. (**a**) Subcellular localization distribution of protein hits with significantly altered abundances. Proteins were categorized based on the information from The Human Protein Atlas and UniProtKB. (**b**) Distribution of direct PPIs reported in the BioGRID database among the 172 regulated proteins reported in the BioGRID database. (**c**) Protein-protein interaction (PPI) network of proteins whose abundances were significantly altered upon W7 treatment. The physical interactions were derived from the BioGRID interaction database. See Supplementary Methods for further information. (**d**) Time-dependent GOLIM4 destabilization induced by W7 treatment (1 μM) in KBM7 cells. Representative images of n = 2 independent measurements. Source data

To identify global proteome changes after W7 treatment, we performed quantitative proteomics in KBM7 cells via the tandem mass tag (TMT) isobaric labeling approach. Out of over 7,800 quantified proteins, 175 displayed altered abundances (log_2_-transformed fold change of >0.25 or <–0.25) after 8 h of treatment (Supplementary Table 2). A notable proportion (116, 66%; adjusted *P* < 0.05) exhibited a substantial reduction in their abundances (Fig. 2a). Functional analysis of these altered proteins revealed a high enrichment of Golgi-localized transmembrane proteins (for example, Golgi integral membrane protein 4 (GOLIM4), GLG1 and TM9SF1–TM9SF4) and proteins involved in glycoprotein biosynthesis (for example, GALNT2/GALNT7, MAN1A1/MAN1A2 and C1GALT1; Fig. 2b). To increase the confidence in the observed phenotype, we investigated if destabilized proteins are known to engage in protein–protein interactions (PPIs) or participate in shared protein complexes. Using the BioGRID database^21^, we created a PPI network between hits whose abundances were significantly altered after W7 treatment. For more than half of the perturbed proteins (97 out of 175, 55%), direct interactions were reported in the literature, which represents significantly more shared edges than expected by random sampling (Extended Data Fig. 4a–c and Supplementary Table 2). Mapping the subcellular localization of proteins within the network illustrated the overall impact on the Golgi proteins as a phenotypic response to W7 treatment. Moreover, we validated the W7-induced time-dependent destabilization of the endosomal cycling protein and trafficking chaperone GOLIM4 (Extended Data Fig. 4d). Collectively, our data indicate that W7 disrupts the Golgi integrity, further supporting morphologic features detected via cell painting assay. The molecular mechanism behind global destabilization of related metabolic (especially glycosylating) and trafficking proteins, however, remained elusive.

To further explore the mechanism of action of W7, we sought to examine the global transcriptional effects after W7 treatment via RNA sequencing (Fig. 2c and Supplementary Table 3). We observed a substantial downregulation of de novo cholesterol biosynthesis genes, such as *HMGCS1*, *LSS*, *DHCR7* and *SQLE*, which encodes one of the two rate-limiting enzymes in this process (Fig. 2c,d). Moreover, key regulators of cholesterol metabolic processes *SREBF2* and *INSIG1* were also downregulated. It is worth noting that similar repression of cholesterol production can be induced by oxysterols such as 25- and 27-hydroxycholesterol (25- and 27-OHC)^22^. These endogenous cholesterol oxidation products suppress the activity of SREBP2 as a transcriptional regulator by hindering its trafficking to the Golgi and thereby preventing its proteolytic processing and maturation^23,24^.

Collectively, multiomics profiling reveals that W7 treatment disrupts cholesterol biosynthesis and affects the cellular abundance of an array of Golgi-associated proteins, indicative of a functional impairment of Golgi homeostasis.

### Cellular efficacy of W7 depends on ER–Golgi PI4P signaling

**Fig. 3:**
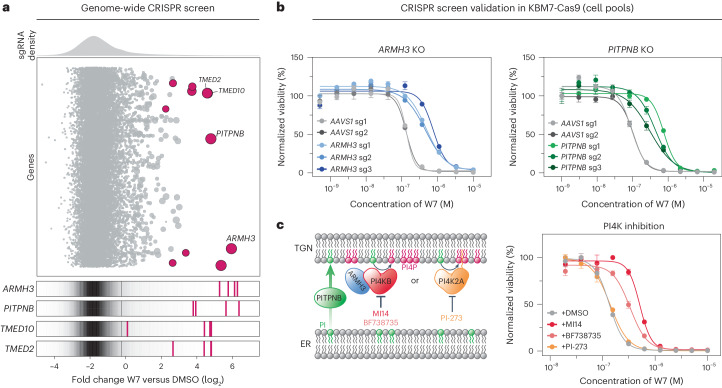
W7 functionally depends on PI4P signaling. **a**, Genome-wide CRISPR–Cas9 knockout screen in W7-treated KBM7 cells constitutively expressing Cas9 (KBM7-Cas9). The bubble plot displays median sgRNA enrichment after W7 treatment over DMSO, and the bubble size represents significance. Only genes with FDR *P* values of ≤0.05 are highlighted. Enrichment of sgRNAs targeting selected hits in comparison to the distribution of all sgRNAs is highlighted separately. See also Supplementary Table 4. **b**, Dose-resolved normalized viability after 72 h of treatment of KBM7-Cas9 cells expressing sgRNAs targeting *ARMH3*, *PITPNB* or *AAVS1* with W7. Data are mean ± s.e.m.; *n* = 3 independent treatments; KO, knockout. **c**, Left, phosphatidylinositol 4-kinases PI4KB and PI4K2A contribute to the functional pool of PI4P in the Golgi. The image illustrates the CRISPR screen hits ARMH3 and PITPNB, along with small-molecule inhibitors of respective phosphatidylinositol 4-kinases. Right, dose-resolved normalized viability of KBM7 cells with W7 in the absence or presence of PI4KB inhibitors MI14 and BF738735 (both 1 μM) or PI4K2A inhibitor PI-273 (1 μM). The cells were cotreated for 72 h. Data are mean ± s.e.m.; *n* = 3 independent treatments. Source data

**Extended Data Fig. 5:**
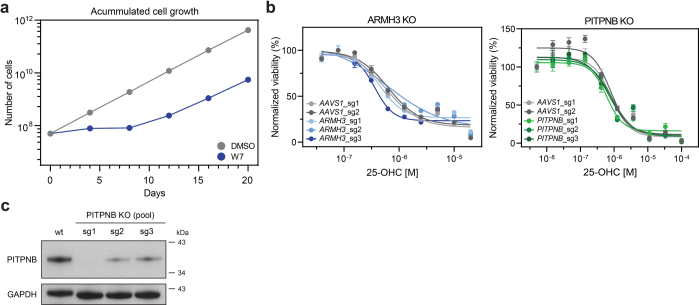
Genome-wide CRISPR/Cas9 resistance screen. (**a**) Accumulated cell growth curve of KBM7-Cas9 treated with DMSO or W7 over 20 days. (**b**) Dose-resolved, normalized viability after 72 h treatment of KBM7-Cas9 cells expressing sgRNAs targeting ARMH3 (blue), PITPNB (green) or AAVS1 locus (grey) with 25-OHC. Mean ± s.e.m.; n = 3 independent treatments. (**c**) PITPNB protein levels in KBM7 pools expressing three different sgRNAs against the gene. Representative images of n = 2 independent measurements. Source data

Next, we wanted to investigate whether the impact on Golgi morphology is cause or consequence of the cellular efficacy of W7. To determine the cellular effectors required for the antiproliferative potential of W7 in leukemia, we performed a genome-wide CRISPR–Cas9 knockout screen in KBM7 cells (Extended Data Fig. 5a and Supplementary Table 4)^25^. Strikingly, top hits included *ARMH3* (until recently *C10orf76*) and *PITPNB* (Fig. 3a). Both genes constitute the molecular machinery required for maintaining PI4P signaling between the ER and other membrane organelles like the *trans*-Golgi network (TGN). While phosphatidylinositol transfer protein-β (PITPNB) mediates the ER-to-TGN transport of phosphatidylinositol proteins^26,27^, Armadillo-like helical domain-containing protein 3 (ARMH3) associates with phosphatidylinositol 4-kinase-β (PI4KB) and has an important role in PI4KB-driven PI4P generation at the Golgi^28,29^. In addition, single guide RNAs (sgRNAs) targeting the components of MCSs involved in lipid transport, like *TMED2* and *TMED10* (ref. ^30^), were also enriched (Fig. 3a). Targeted CRISPR-induced inactivation of *PITPNB* and *ARMH3* in KBM7 cell pools confirmed their requirement for W7 efficacy (Fig. 3b). PI4P is generally synthesized at the TGN by type 2 (for example, PI4K2A) and type 3 (for example, PI4KB) phosphatidylinositol 4-kinases^31,32^. Interestingly, chemical inhibition of the type 2 kinase PI4K2A with PI-273 did not have an impact on W7-induced cytotoxicity in KBM7 cells. PI4KB inhibitors MI14 and BF738735, however, effectively reversed the drug-induced effect (Fig. 3c), underscoring the functional dependence of the cellular efficacy of W7 on PI4P produced by the type 3 kinase and supporting the CRISPR screening data. Apart from its active role in regulating membrane trafficking, the intrinsic gradient of PI4P (high within the TGN and low within the ER) also drives the nonvesicular transport of lipids, especially cholesterol, at MCSs. Several endogenous metabolites, such as oxysterols, are involved in phosphatidylinositol metabolism but also contribute to the regulation of cholesterol homeostasis. Given that W7 induced transcriptional repression of cholesterol biosynthesis (Fig. 2c,d), we wanted to distinguish it from oxysterols like 25-OHC. Therefore, we probed the cellular efficacy of 25-OHC in *PITPNB*- and *ARMH3*-knockout backgrounds. In both cases, depletion of PITPNB and ARMH3 did not affect 25-OHC cytotoxicity in KBM7 cells (Extended Data Fig. 5b,c).

Together, our findings show that the efficacy of W7 functionally depends on PI4P signaling between the ER and the Golgi. Given the essential role of PI4P in maintaining Golgi function, we reason that the observed impact on Golgi homeostasis is causative for the leukemic vulnerability to W7. Hence, we anticipated that the direct protein target(s) of W7 would be involved in Golgi-associated processes.

### Orpinolide (W7) targets the oxysterol-binding protein OSBP

**Fig. 4:**
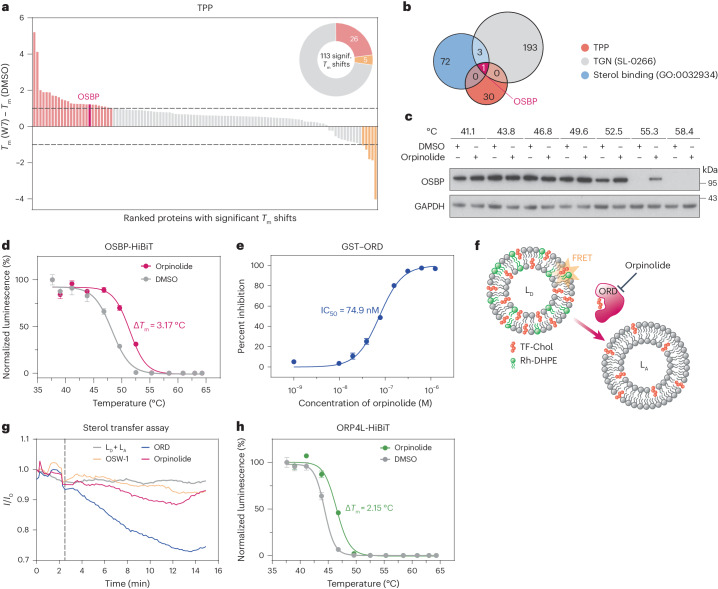
Orpinolide (W7) directly targets OSBP and inhibits its sterol transport function. **a**, TPP. Among 113 significantly altered melting profiles, 31 hits show a Δ*T*_m_ exceeding 1 °C (highlighted). See also Supplementary Table 5. **b**, Overlay of TPP hits with TGN-localized (UniProtKB SL-0266) and sterol-binding (GO:0032934) proteins reveals OSBP as the putative target of orpinolide. **c**, Orpinolide (5 μM, 2 h) stabilizes endogenous OSBP in intact KBM7 cells. Representative images of *n* = 3 independent measurements are shown. **d**, Orpinolide (1 μM, 4 h) stabilizes HiBiT-tagged OSBP in intact KBM7 cells. Data are mean ± s.e.m.; *n* = 3 independent experiments; *n* = 3 technical replicates. **e**, Fluorescence polarization competition assay. Orpinolide displaces 22-NDB-cholesterol from purified GST-ORD. Data are mean ± s.e.m.; *n* = 3 independent experiments; *n* = 3 technical replicates; IC_50_, half-maximal inhibitory concentration. **f**, FRET-based in vitro cholesterol transfer assay. **g**, Orpinolide (500 nM) disrupts the transfer of TF-Chol from the donor (L_D_; 16 μM) to the acceptor (L_A_; 16 µM) vesicles mediated by ORD (500 nM). The OSBP inhibitor OSW-1 (500 nM) was used as a positive control. Data are representative of *n* = 2 independent experiments. **h**, Orpinolide-induced (1 μM, 4 h) thermal stabilization of HiBiT-tagged ORP4L in intact KBM7 cells. Data are mean ± s.e.m.; *n* = 2 independent experiments; *n* = 3 technical replicates. Source data

**Extended Data Fig. 6:**
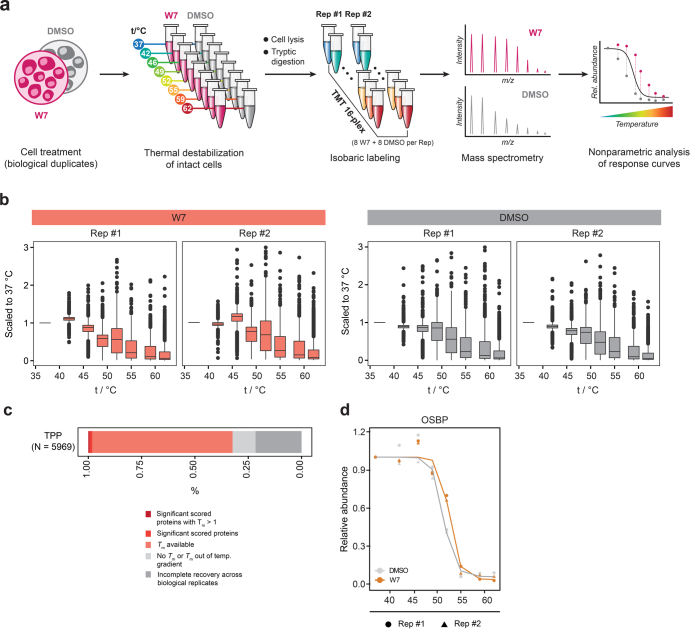
Thermal proteome profiling. (**a**) Overview of the experimental setup. Drug/vehicle treatment and 8-temperature destabilization were performed in biological duplicates. Significant shifts in drug-induced melting profiles were identified with NPARC. (**b**) Distribution of TPP data after scaling each protein to the abundance signal in the lowest temperature condition (37 °C), shown for individual W7/DMSO-treated replicates (n = 2, biological replicates). The solid line in the box plots represents the median, box limits show the interquartile range (IQR) and its whiskers 1.5 x IQR. Outliers are represented as block dots. (**c**) Overview of TPP results represented as fraction of proteins in respect to individual analysis steps. (**d**) Melting profiles for the W7 hit OSBP, detected by NPARC (adj. P-value = 0.00496; n = 2, biological replicates). Source data

**Extended Data Fig. 7:**
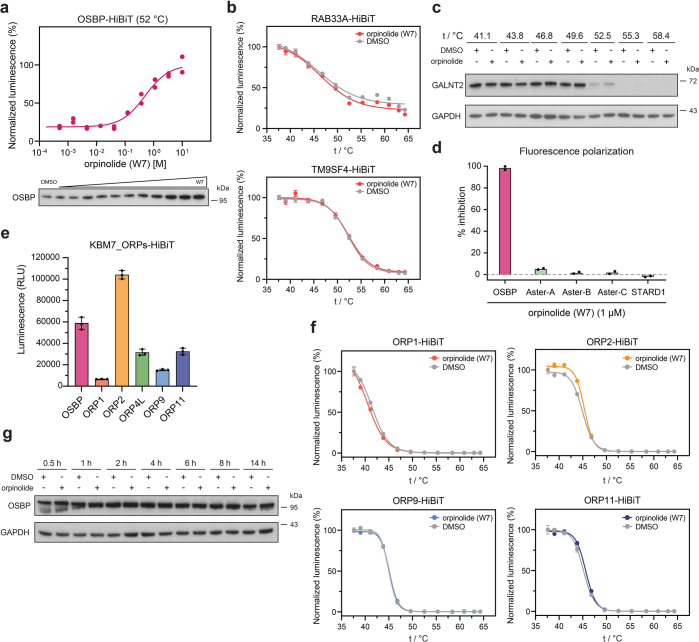
Oxysterol-binding protein OSBP is a putative target of orpinolide (W7). (**a**) Top: Dose-dependent stabilization of HiBiT-tagged OSBP at 52 °C in intact KBM7 cells (4 h treatment). n = 2 technical replicates; representative of n = 2 independent experiments. Bottom: Endogenous OSBP levels in KBM7 after dose-resolved orpinolide treatment and thermal destabilization at 52 °C. Representative image of n = 2 independent measurements. (**b**) Orpinolide (5 μM, 2 h) does not thermally stabilize HiBiT-tagged RAB33A (up) or TM9SF4 (down) in intact KBM7 cells. Mean ± s.e.m.; n = 3 technical replicates; representative of n = 3 independent experiments. (**c**) Orpinolide (5 μM, 2 h) does not thermally stabilize endogenous GALNT2 in intact KBM7 cells. Representative images of n = 3 independent measurements. (**d**) Fluorescence polarization competition assay measuring the displacement of 22-NDB-cholesterol from purified GST-OSBP(ORD), Aster-A/B/C and STARD1 upon orpinolide treatment (1 μM). n = 2 independent measurements. (**e**) Expression levels of HiBiT-tagged ORPs in KBM7 cells. Mean ± s.e.m.; n = 3 independent measurements. (**f**) Orpinolide (1 μM, 4 h) does not thermally stabilize HiBiT-tagged ORP1, ORP2, ORP9 or ORP11 in intact KBM7 cells. Mean ± s.e.m.; n = 3 technical replicates; representative of n = 2 independent experiments. (**g**) Effect of orpinolide treatment (1 μM) on OSBP protein levels in KBM7 cells. Representative image of n = 2 independent measurements. Source data

The functional requirement of PI4P signaling led us to surmise that W7 might interfere with the sterol transport at ER–Golgi contact sites. To identify cellular targets of W7, we performed TPP in KBM7 cells. Using multiplexed quantitative proteomics, TPP allows the assessment of proteome-wide changes in protein stability following interactions with small molecules^33^. Importantly, the distinct changes in the melting profiles of proteins are indicative of putative drug targets. After initial treatment with DMSO or W7, intact KBM7 cells were subjected to thermal destabilization with an eight-temperature gradient (37–62 °C). Following mild cell lysis, the samples were processed through a standard quantitative proteomics workflow via TMT isobaric labeling (Extended Data Fig. 6a). Subsequent nonparametric analysis of response curves (NPARC)^34^ revealed 4,037 melting profiles, of which 113 were significantly altered (adjusted *P* < 0.01; Extended Data Fig. 6b–d, Supplementary Fig. 1 and Supplementary Table 5). These included both stabilization (26) and destabilization (5) profiles with a change in melting temperature (Δ*T*_m_) exceeding 1 °C (Fig. 4a and Extended Data Fig. 6c). Considering the information embedded in the experimental data acquired hitherto, we overlaid the 31 discrete hits with the TGN-localized (UniProtKB SL-0266) and sterol-binding (GO:0032934) proteins. This intersection revealed OSBP as the putative target of W7 (Fig. 4b and Extended Data Fig. 6d). As a major lipid transporter at ER–Golgi contact sites, OSBP uses the metabolic energy of PI4P to exchange cholesterol as well as PI4P between the membranes^35,36^. We confirmed drug binding to OSBP through two versions of a cellular thermal shift assay (CETSA), including immunoblotting of endogenous OSBP in KBM7 cells (Fig. 4c) and a bioluminescence readout in KBM7 cells overexpressing HiBiT-tagged OSBP (Fig. 4d and Extended Data Fig. 7a). It is worth noting that we detected additional Golgi-associated proteins, such as the Ras-related GTPase RAB33A, the transmembrane protein TM9SF4 and the GalNAc transferase GALNT2, in our TPP data (Supplementary Table 5). However, thermal stabilization could not be validated in targeted CETSA experiments, indicating that they are likely false positives identified in the TPP assay (Extended Data Fig. 7b,c). In summary, TPP and CETSA experiments point to OSBP as a direct protein target of W7, prompting us to term the compound orpinolide henceforth.

In vitro fluorescence polarization assays with a recombinant glutathione *S*-transferase (GST)-tagged sterol-binding domain of OSBP (OSBP-related domain or ORD)^37^ indicated that orpinolide engages OSBP in the nanomolar range and that binding is selective over other sterol transporters^38^^,39^ (Fig. 4e and Extended Data Fig. 7d). Furthermore, we evaluated the effect of orpinolide on the cholesterol transport function of OSBP. To this end, we used a fluorescence resonance energy transfer (FRET)-based in vitro assay involving the transfer of a fluorescent cholesterol analog (TF-Chol) between synthetic donor (L_D_) and acceptor (L_A_) liposomes. The L_D_ liposomes contained a rhodamine B-labeled phospholipid (Rh-DHPE) in conjunction with TF-Chol, thus forming a FRET pair (Fig. 4f). Facilitated by recombinant GST-ORD, the transfer of TF-Chol between L_D_ and L_A_ resulted in a reduction in the measured FRET signal (Fig. 4g, blue). Notably, the addition of orpinolide rescued the reduction in FRET signal, indicating a hindered TF-Chol transport as a consequence of GST-ORD inhibition (Fig. 4g, pink). This closely resembled the effect of OSW-1 (Fig. 4g, orange), a previously characterized inhibitor of OSBP^40^, demonstrating the consistency of the orpinolide action.

OSBP is the prototypical member of the OSBP-related protein (ORP) family, dual lipid transporters at MCSs characterized by the possession of conserved ORD domains^41^. To profile orpinolide specificity within this protein family, we overexpressed several different ORPs (ORP1, ORP2, a full-length variant of ORP4 (ORP4L), ORP9 and ORP11) as HiBiT fusions (Extended Data Fig. 7e) and assessed orpinolide binding via HiBiT CETSA in intact KBM7 cells. In particular, we focused on ORPs known for binding cholesterol (based on reported in vitro or in vivo data) and/or Golgi localization^42^. We observed that orpinolide stabilized the closely related ortholog ORP4L, albeit to a lower degree (Fig. 4h). By contrast, none of the other tested ORPs were thermally stabilized in the presence of the compound (Extended Data Fig. 7f). Of note, although OSBP and other ORPs were detected in expression proteomics (Fig. 2a), their relative abundances were unchanged after compound treatment (Supplementary Table 2), which we could also confirm in the case of OSBP via immunoblotting (Extended Data Fig. 7g). Collectively, these data establish OSBP/ORP4L as cellular targets of orpinolide.

### OSBP is the phenotypically relevant target of orpinolide

**Fig. 5:**
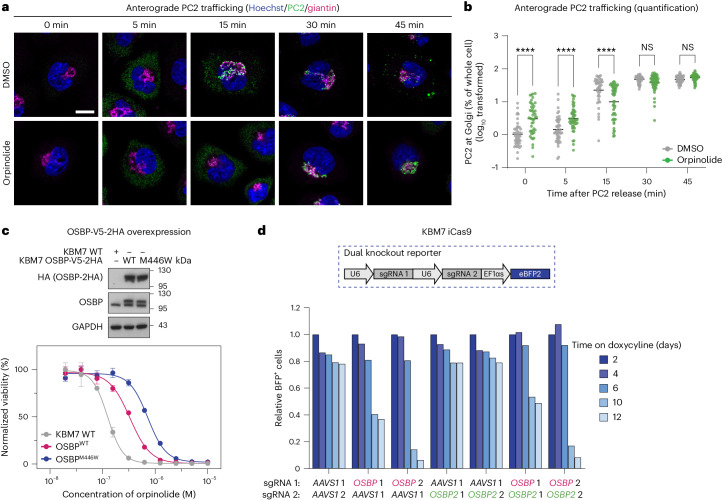
Cellular efficacy of orpinolide arises from functional OSBP inhibition. **a**, Scanning confocal microscopy analysis of the effects of orpinolide treatment (1 μM) on PC2 trafficking in RCS cells. PC2 secretion was synchronized for 4 h at 40 °C before being released by incubation at 32 °C for the indicated duration. Representative images show PC2 localization (488, green), the Golgi area (giantin, 568, purple) and the nuclei (Hoechst, blue); scale bar, 10 μm. **b**, Quantification of PC2 localization to the Golgi normalized to total PC2 per cell. The graph shows one point per cell from three independent experiments, with between 42 and 58 cells per condition. The data are log_10_-transformed and were analyzed by two-way ANOVA, with *P*_interaction_ < 0.0001 and *P*_treatment factor_ < 0.01. A Šídák multiple comparison test compared DMSO against orpinolide at each time point; ****P < 0.0001; NS, not significant (*P* = 0.4783, 30 min; *P* = 0.9126, 45 min). **c**, Top, expression levels of endogenous OSBP and V5-2HA-tagged OSBP variants (wild-type or M446W mutant) in both wild-type KBM7 cells and KBM7 cells overexpressing these fusions. Bottom, dose-resolved normalized viability after 72 h of treatment of wild-type KBM7 cells and KBM7 cells overexpressing V5-2HA-tagged OSBP variants (wild-type or M446W mutant) with orpinolide. Data are mean ± s.e.m.; *n* = 3 independent treatments; WT, wild type. **d**, CRISPR-based dropout experiment in doxycycline-inducible Cas9 KBM7 (KBM7 iCas9) cells transduced with a BFP-based dual-knockout reporter. The reporter plasmid carries a combination of sgRNAs targeting *OSBP*, *OSBP2* or *AAVS1* to yield single or dual *OSBP*/*OSBP2* knockout. The bar plots depict relative BFP^+^ cells over 12 days of doxycycline treatment normalized to day 2 for each individual reporter system. Source data

**Extended Data Fig. 8:**
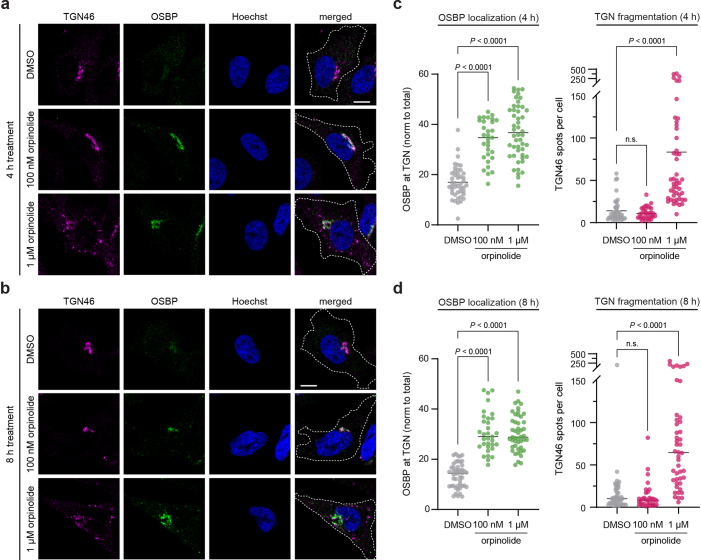
Orpinolide-induced OSBP inhibition triggers alterations in the morphology and functionality of the Golgi apparatus. (**a**, **b**) Scanning confocal microscopy analysis of the effects of orpinolide treatment on OSBP localization and *trans*-Golgi morphology in HeLa cells. Cells were treated with orpinolide (100 nM or 1 μM) or DMSO for 4 h (**a**) or 8 h (**b**) and immunostained with antibodies against OSBP (488, green) and TGN46 (568, purple) while the nuclei were labelled with Hoechst (blue). The scale bar is 10 μm. Dotted line indicates cell outline. (**c**, **d**) Quantification of OSBP localization to the Golgi as identified by TGN46 (left) and *trans*-Golgi fragmentation measured by number of TGN46 spots per cell (right) after 4 h (**c**) and 8 h (**d**) orpinolide treatment. OSBP fluorescence intensity was measured in TGN46 region of interest (ROI) and normalized to total cellular OSBP. The graphs show one point per cell from at least 2 independent experiments for a total of 44 (DMSO; n = 3), 31 (100 nM orpinolide; n = 2) and 48 cells (1 μM orpinolide; n = 3) (**c**); 47 (DMSO; n = 3), 33 (100 nM orpinolide; n = 2) and 46 cells (1 μM orpinolide; n = 3) (**d**). Line indicates mean. One-way ANOVA was performed on log10 transformed data (see Supplementary Fig. 2a); *P* < 0.0001 with Dunnett’s multiple comparisons test; n.s. *P* = 0.8432 (**c**); n.s. *P* = 0.7773 (**d**). Source data

**Extended Data Fig. 9:**
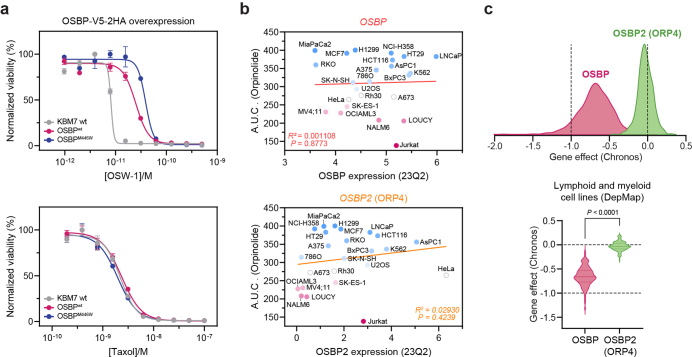
Genetic dependencies of cancers on OSBP and OSBP2. (**a**) Dose-resolved, normalized viability after 72 h treatment of wild type KBM7 cells and KBM7 cells overexpressing V5-2HA-tagged OSBP variants (wild type or M446W mutant) with OSW-1 (top) or paclitaxel (bottom). Mean ± s.e.m.; n = 3 independent treatments. (**b**) Comparison of the sensitivity of various cancer cell lines to orpinolide with the expression levels of *OSBP* and *OSBP2* (DepMap 23Q2). The compound sensitivity is represented as area under the curve (A.U.C.) calculated from 10-concentration-point dose-response curves after 72 h drug treatment (see Extended Data Fig. 3). (**c**) Top: Distribution of OSBP and ORP4 (*OSBP2*) deletion effects across 1078 cancer cell lines from the DepMap 22Q4 CRISPR data. Bottom: Distribution of OSBP and ORP4 (*OSBP2*) deletion effects across myeloid and lymphoid cancer cell lines from the DepMap 22Q4 CRISPR data. Wilcoxon matched-pairs signed ranked test was performed on the data. Source data

Our in vitro studies clearly demonstrate that orpinolide binds to OSBP and inhibits sterol transport between synthetic liposomes. Next, we aimed to further investigate the functional repercussions of orpinolide treatment in living cells. To this end, we first monitored the effects of orpinolide on OSBP localization and overall Golgi integrity using immunofluorescence microscopy. Short treatment (4 h) of HeLa cells with orpinolide disrupted the cytoplasmic distribution of OSBP, even at concentrations as low as 100 nM. Instead, OSBP predominantly localized within the TGN, as evidenced by a high degree of colocalization with the TGN46 marker (Extended Data Fig. 8a,b). In line with the phenotype observed in expression proteomics (Fig. 2a,b), the relocalization of OSBP was accompanied by partial fragmentation of the Golgi, which became particularly evident at higher orpinolide concentrations and later time points (Extended Data Fig. 8c,d and Supplementary Fig. 2a). To investigate whether OSBP inhibition affected the anterograde transport of cargo between the ER and the Golgi, we examined the trafficking of type 2 procollagen (PC2) in rat chondrosarcoma (RCS) cells (Fig. 5a,b and Supplementary Fig. 2b). PC2 accumulates in the ER when incubated at 40 °C but is released from the organelle after lowering the temperature to 32 °C (time 0 min), transported toward the Golgi and eventually secreted. Notably, orpinolide treatment substantially delayed the trafficking of PC2, as indicated by a reduction in the amount of PC2 observed at the Golgi at the 15-min time point. Furthermore, orpinolide also delayed the departure of PC2 from the Golgi, leading to higher retention of PC2 in the Golgi at the 45-min time point. Together, these data underscore the functional effect of orpinolide-induced OSBP inhibition on the normal functioning of the Golgi. In an attempt to correlate the observed functional outcomes following OSBP inhibition with the antiproliferative effect of orpinolide in leukemia, we investigated whether OSBP overexpression could confer resistance to orpinolide. We introduced V5-2HA-tagged OSBP into KBM7 cells, along with a dysfunctional OSBP variant bearing a M446W mutation in its ligand-binding ORD domain (Fig. 5c, top). This mutation has previously been recognized for diminishing the antiproliferative efficacy of various OSBP inhibitors, including OSW-1 (refs. ^40,43^). Overexpression of both wild-type OSBP (OSBP^WT^) and mutant OSBP (OSBP^M446W^) rendered KBM7 cells less responsive to orpinolide treatment (Fig. 5c, bottom), confirming that the antileukemic properties of orpinolide arise from OSBP inhibition. As expected, we observed a similar shift with OSW-1, but not with the negative control paclitaxel (Extended Data Fig. 9a).

**Extended Data Fig. 10:**
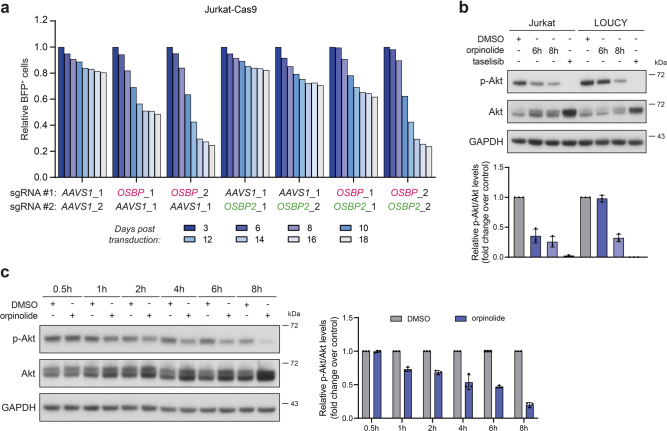
Functional inhibition of OSBP underlies the cellular efficacy of orpinolide. (**a**) CRISPR-based dropout screen in Jurkat cells constitutively expressing Cas9, transduced with a BFP-based dual knockout reporter. The reporter plasmid carries a combination of sgRNAs targeting *OSBP*, *OSBP2* or *AAVS1* locus to yield single or dual OSBP/ORP4 knockout. The bar plots depict relative BFP^+^ cells over 18 days, normalized to day 3 post transduction for each individual reporter system. (**b**) Top: Effect of orpinolide treatment (5 μM) on p-Akt and Akt protein levels in Jurkat and LOUCY cells. Phosphoinositide 3-kinase (PI3K) inhibitor taselisib (0.5 μM, 8 h treatment) was used as a positive control. Representative image of n = 3 independent experiments. Bottom: Quantification of the relative p-Akt/Akt protein levels normalized to the DMSO controls. Mean ± SD; n = 3 independent experiments. (**c**) Left: Effect of orpinolide treatment (5 μM) on p-Akt and Akt protein levels in Jurkat cells at different time points. Representative image of n = 3 independent experiments. Right: Quantification of the relative p-Akt/Akt protein levels normalized to the DMSO controls. Mean ± SD; n = 3 independent experiments. Source data

Previous studies have implicated the dysregulation and/or aberrant expression of various members of the ORP family with the development of cancer^44,45^. OSBP and ORP4L have, for example, been revealed as targets of several NPs displaying antiproliferative effects in diverse cancer cell lines^40^. Moreover, the proliferation and survival of immortalized and cancerous cell lines, especially T cell-derived lymphoblastic leukemia, seemingly require the expression of ORP4 (refs. ^46,47^). Intriguingly, despite this, a comparison between the sensitivities of various cancer cell lines to orpinolide (Extended Data Fig. 3) and the mRNA expression levels of *OSBP* or ORP4-encoding *OSBP2* did not reveal significant correlations (Extended Data Fig. 9b). We thus wanted to investigate if functional interference with OSBP, ORP4 or both recapitulates the orpinolide-induced vulnerability phenotype of leukemia cells. We therefore analyzed publicly available genome-wide CRISPR–Cas9 screens across 1,078 cell lines, which unveiled the ORP4-encoding *OSBP2* as a nonessential gene. By contrast, a distinct dependency on *OSBP* was observed in multiple cell lines, including cells originating from myeloid or lymphoid lineages (Extended Data Fig. 9c)^48^. To validate these pooled screening results in an arrayed format in the context of leukemia, we conducted a CRISPR-based dropout experiment in KBM7 and Jurkat cells by inducing either single or dual *OSBP*/*OSBP2* knockout(s). By monitoring the decrease of the sgRNA (blue fluorescent protein, BFP) positive cells over time, our results suggest that the survival of KBM7 cells solely depends on *OSBP* without notable contribution of *OSBP2* (Fig. 5d). A similar effect of OSBP inactivation was observed in Jurkat cells (Extended Data Fig. 10a). Dysregulation of cellular PI4P and cholesterol levels not only affects Golgi function but also influences the output of cellular signal transduction networks, such as the oncogenic PI3K–AKT–mTOR signaling axis, an established leukemic vulnerability^17,49–51^. When evaluating the effects of orpinolide on AKT signaling, we observed a marked decrease of AKT phosphorylation in Jurkat and LOUCY cells during extended compound treatment (Extended Data Fig. 10b,c). Downregulation of AKT signaling can therefore be considered a downstream consequence of OSBP inhibition, hence rationalizing why leukemia cells, especially T cell-derived ALL, are more sensitive to orpinolide treatment.

In summary, we uncover that orpinolide interferes with the sterol transport at ER–Golgi MCSs by selectively targeting OSBP and ORP4L over other ORPs. Importantly, we disentangle the cellular efficacy of orpinolide by showing that it predominantly emanates from targeting OSBP, reaffirming it as a putative therapeutic target in the context of leukemia.

## Discussion

Here, we assessed the antileukemic properties of a focused compound collection derived from the core withanolide scaffold and identified orpinolide among several vinylogous urethane analogs with pronounced antiproliferative effects. To dissect its mode of action, we integrated quantitative proteomics, transcriptomics and functional genomics screens and revealed that orpinolide treatment leads to functional impairment of Golgi homeostasis by targeting and inhibiting OSBP. Serving as a lipid transporter at ER–Golgi MCSs, OSBP uses the metabolic energy of PI4P to drive the transport of ER-produced cholesterol to the Golgi. At the same time, it helps maintain the intrinsic PI4P gradient between membranes by transferring PI4P back to the ER where it is rapidly dephosphorylated^35,36^. Hence, OSBP is an essential component of nonvesicular lipid transport at MCSs. After profiling the specificity of orpinolide against several other members of the ORP family, we identified ORP4 as its additional target. Like OSBP, ORP4 is involved in sterol/PI4P countertransport^46^.

Orpinolide represents a viable withanolide-derived inhibitor of OSBP/ORP4 activity. OSBP/ORP4 are also bound by oxysterols, including 25-OHC. However, our results clearly differentiate the effect of orpinolide from 25-OHC where the functional dependence of orpinolide on active PI4P signaling was not recapitulated by 25-OHC. The promiscuity of 25-OHC, which interacts with a plethora of sterol-sensing proteins^52,53^, could serve as an explanation for this difference, but definitive elucidation will require further investigation. Along similar lines, several bioactive NPs have previously been reported as OSBP/ORP4 modulators and have collectively been referred to as ‘ORPphilins’^40^. This includes compounds such as OSW-1 and schweinfurthin A. Of note, their molecular mechanism of OSBP modulation appears to be highly diverse. For instance, OSW-1 treatment induced proteasome-dependent degradation of OSBP via an entirely elusive mechanism. By contrast, orpinolide has no consequence on OSBP stability or abundance. Furthermore, although both orpinolide and schweinfurthin A cause OSBP to localize within the TGN, OSW-1 has been shown to induce dispersal of OSBP. Nevertheless, a recent study highlighted the functional dependency of OSW-1 in HeLa cells on PITPNB, ARMH3 and PI4KB^54^, indicating that the genetic factors governing the efficacy of different OSBP-targeting compounds may be shared. The specific and individual molecular consequences (including quantification of ensuing changes in sterol/lipid levels) prompted by different compounds that bind to OSBP/ORP4 will hence require further elucidation. Caution in interpreting cellular phenotypes will, however, have to be exercised as the off-target profile of ORPphilins and related molecules are, for the most part, elusive. One reason for this shortcoming is the time- and resource-intensive synthesis or isolation of these NPs. By contrast, orpinolide is accessible by means of a short and efficient synthesis sequence^15^.

Several studies have addressed the function of ORP4 in leukemia. Previous work has implied ORP4 as an essential driver of T cell-derived ALL proliferation and found it to be overexpressed in malignant compared to benign T cells^47^. Initially, these observations provided an intriguing hypothesis for the observed antileukemic efficacy of orpinolide as a selective dual inhibitor of OSBP/ORP4. Arrayed gene disruption experiments of OSBP and ORP4, either individually or in combination, however, clearly indicate that OSBP represents the predominant dependency. This finding is consistent with genome-scale CRISPR–Cas9 screening data that are publicly available in DepMap^48^. Our data suggest that, even when assessed in combination with *OSBP* knockout, ORP4 disruption prompts no measurable additional fitness defect. Supported by OSBP rescue data, we hence surmise that the antiproliferative consequences of orpinolide are conveyed by OSBP inhibition, even though we cannot rule out the contribution of additional factors at high orpinolide concentrations. Notably, through orchestrating the transport of PI4P between the ER and the plasma membrane, several members of the ORP family, including OSBP, have been shown to sustain hyperactive PI3K/AKT signaling^55,56^, a known vulnerability in leukemia cells, especially T cell-derived ALL cell lines^57,58^. Indeed, cellular treatment with orpinolide led to attenuated AKT signaling, thus providing a mechanistic rationale for its antileukemic activity. Likewise, this could explain the observed sensitivity of Ewing sarcoma cell lines, which are also frequently reliant on an aberrantly activated PI3K–AKT–mTOR signaling pathway^59,60^. Of note, the kinetics of the observed orpinolide effects on AKT signaling suggest that it is a secondary effect that occurs downstream of OSBP inhibition and subsequent changes in sterol/PI4P levels. OSBP-mediated cholesterol transport to lysosomes has also been implicated in sustaining aberrant mTORC1 signaling, particularly in disease models of Niemann–Pick disease, which is caused by loss of the lysosomal cholesterol transporter NPC1 (ref. ^61^). The significance of the altered PI3K–AKT–mTOR pathway in relation to the antileukemic effects of orpinolide and its potential applicability to other cancer models reliant on this pathway will be the subject of future research.

Collectively, the presented data establish orpinolide as a probe-like small molecule that can be used to investigate cholesterol transport by OSBP and ORP4. Our data also reaffirm sterol transport at MCSs as a druggable metabolic dependency in leukemia cells that will motivate future translational efforts.

## Methods

### Cell lines and cell culture

KBM7 cells (obtained from T. Brummelkamp, Netherlands Cancer Institute) were grown in IMDM supplemented with 10% fetal bovine serum (FBS) and 1% penicillin/streptomycin (Pen/Strep). MOLT4, Jurkat, LOUCY and P12-Ichikawa cells were obtained from the laboratory of J. E. Bradner (Dana-Farber Cancer Institute). All other cell lines were purchased from ATCC unless otherwise stated. 768-O, A375, A673, BxPC3, DU145, H1299, HT29, Jurkat, K562, LNCaP, LOUCY, MCF7, MOLT4, MV4-11, NALM6, NCI-H358, OCIAML3, P12-Ichikawa, Rh30 and SK-E-S1 cells were grown in RPMI supplemented with 10% FBS and 1% Pen/Strep. 293T, AsPC1, HCT116, HeLa, MiaPaCa2, RKO, U2OS and Lenti-X 293T lentiviral packaging cells were grown in DMEM supplemented with 10% FBS and 1% Pen/Strep. RPE-1 and SK-N-SH cells were maintained in DMEM/F-12 supplemented with 10% FBS and 1% Pen/Strep. RCS cells (a gift from C. Settembre, Telethon Institute of Genetics and Medicine) were grown in DMEM supplemented with 10% FBS, 1 mM sodium pyruvate, 2 mM glutamine and 1% Pen/Strep. KBM7 and Jurkat cells constitutively expressing Cas9 were generated as previously described^62^. KBM7 iCas9 cells were a gift from J. Zuber (Research Institute of Molecular Pathology). Nonmalignant PBMCs were isolated from the peripheral blood of a healthy adult volunteer (purchased from the local transfusion service, Red Cross Austria) via density gradient centrifugation (Lymphoprep, Stemcell Technologies) and were maintained in RPMI supplemented with 10% FBS and 1% Pen/Strep. Cell lines were grown in a humidified incubator at 37 °C and 5% CO_2_ and were regularly tested for mycoplasma contamination.

### Plasmids, cloning and protein expression constructs

*OSBP*, *ORP2*, *ORP4L*, *ORP9* and *ORP11* cDNAs were custom synthesized by Twist Biosciences to include a C-terminal V5 tag and *attB* sites and were cloned into pDONR221 (Thermo Fisher, 12536017). *ORP1*, *TM9SF4* and *RAB33A* cDNAs were purchased in a Gateway-compatible pGenDONR vector from GenScript. A pLEX306-HiBiT destination vector, a kind gift from M. Erb (The Scripps Research Institute), was used for lentiviral expression of C-terminally tagged HiBiT fusions^63^. The M446W mutation was introduced into pDONR221-OSBP-V5 via Q5 site-directed mutagenesis (New England Biolabs, E0552) using the following primers: 5′-GCCCTTGTCCTGGCTTCAGCGCC-3′ (forward) and 5′-TCATTAAAGTTTACCGGCATGG-3′ (reverse). A pLEX-2HA-P2A-Puro destination vector, kindly provided by the laboratory of A. Bergthaler (Medical University of Vienna), was used for lentiviral expression of C-terminally tagged OSBP^WT^/OSBP^M446W^–V5-2HA fusions. *ARMH3*-, *PITPNB*-, *OSBP*- and *OSBP2*-targeting sgRNAs were designed using the Vienna Bioactivity CRISPR score portal^64^ and were cloned into LentiGuide-Puro (Addgene, 52963) or into pLenti-U6-sgRNA1-U6-sgRNA2-EF1αs-eBFP2 (a kind gift from J. Zuber (Research Institute of Molecular Pathology)) following standard protocol^65,66^. Human ASTER domains of Aster-A_359–547_, Aster-B_364–552_ and Aster-C_318–504_ were subcloned into a pGEX-6p-2rbs vector, thus introducing the cloning artifact ‘GPLGS’^38^. The pGEX-6p-1-GST-OSBP_377–807_ plasmid was obtained from Genscript. The pET22b-His6-STARD_166–284_ plasmid was a gift from J. H. Hurley (University of California)^67^. All generated plasmids and sgRNA sequences are listed in the Supplementary Table 7. The sequences of ORP cDNAs and synthesized fragments are listed in Supplementary Table 6.

### Lentivirus production and transduction

Lenti-X 293T cells (at approximately 80% confluency) were cotransfected with the target vector, lentiviral psPAX2 helper (Addgene, 12260) and pMD2.G envelope (Addgene, 12259) using polyethyleneimine (PEI MAX molecular weight 40,000 Da, Polysciences, 24765-100) as previously described^68^. Viral supernatant was collected after 72 h and cleared of cellular debris by filtration through a 0.45-µm PES filter. Cells were transduced with respective virus via spinfection (2,000 rpm, 1 h, 37 °C) in the presence of polybrene (8 μg ml^–1^; Santa Cruz, SC-134220). For generation of *ARMH3* and *PITPNB* knockouts in KBM7-Cas9 cells, cells were selected with puromycin (1 μg ml^–1^; Gibco, A1113803) 2 days after transduction for a total of 5 days, after which the knockout pools were validated via western blotting. Similarly, KBM7 cells overexpressing HiBiT- or 2HA-tagged proteins of interest were selected with puromycin 2 days after transduction for a total of 5 days and validated via luminescence measurements or western blotting.

### Phenotypic screening of the withanolide-inspired compound collection

The 52-membered withanolide-inspired compound collection (Extended Data Fig. 1a, Supplementary Table 1 and Supplementary Information for data on orpinolide purity) was designed and synthesized as previously reported^15^. The compounds were printed onto 384-well plates (Corning, 3570) in eight concentration points (10, 3, 1, 0.3, 0.1, 0.03, 0.01 and 0.003 μM) and in biological triplicates. DMSO was used as a negative control, while staurosporine (1 μM) was used as a cytotoxic positive control. Both controls were seeded in multiple wells and in all of the screened plates.

Wild-type cells (K562, KBM7, MV4-11, OCIAML3, NAML6, Jurkat, LOUCY, MOLT4 and P12-Ichikawa) were seeded at a density of 1,000 cells per well (50-μl assay volume) with a Liquid Handler Multidrop Combi. Cell viability was assessed after 72 h with a CellTiter-Glo assay (Promega, G7573). Survival curves and area under the curve values were determined in GraphPad Prism (v.9.5.1) by interpolation of a sigmoidal standard curve. Each data point was normalized to the mean luminescence of DMSO controls.

### Cell viability assay

Cells were seeded in 96-well plates at the density of 5,000 cells per well and treated with DMSO or drug (1:2 or 1:3 serial dilution; ten different concentrations) for 72 h in biological triplicates (adherent cells were left incubating for 12 h before treatment). Cell viability was assessed with a CellTiter-Glo assay (Promega, G7573) according to manufacturer’s protocol. Luminescence signal was measured on a Multilabel Plate Reader Platform Victor X3 model 2030 (PerkinElmer). Survival curves and EC_50_ values were determined in GraphPad Prism (v.9.5.1) by fitting a nonlinear regression curve or interpolation of a sigmoidal standard curve. Each data point was normalized to the mean luminescence of DMSO controls or the lowest drug concentration.

### Cell painting assay

The cell painting assay was performed in a 384-well plate format in U2OS cells in triplicates. Plate preparation, image acquisition, subsequent cellular feature extraction (579 in total) and compilation of phenotypic profiles were performed as previously described^69^. The phenotypic profile of a compound is determined as the list of *z* scores of all features (calculated relative to the median of DMSO controls and defined by how many median absolute deviations the measured value is away from the median of the controls) for one compound. Additionally, an induction value was determined for each compound as the fraction of significantly changed features in percentage (eq. (1)).

1 $${{\mathrm{Induction}}}\,\left( \% \right)=\frac{{{\mathrm{Number}}}\,{{\mathrm{of}}}\,{{\mathrm{features}}}\,{{\mathrm{with}}}\,{{\mathrm{abs}}}.{{\mathrm{values}}} > 3}{{{\mathrm{Total}}}\,{{\mathrm{number}}}\,{{\mathrm{of}}}\,{{\mathrm{features}}}}$$

Similarities of phenotypic profiles (or biosimilarity) were calculated from the correlation distances between two profiles (biosimilarity = 1 – correlation distance; https://docs.scipy.org/doc/scipy/reference/generated/scipy.spatial.distance.correlation.html), and the compounds with the most similar profiles were determined from a set of 3,000 reference compounds that was also measured in the assay. UMAP analysis was performed as previously reported^70^.

### Western blotting

Collected cell pellets were washed with PBS and lysed in RIPA buffer (50 mM Tris-HCl (pH 8.0), 150 mM NaCl, 1% Triton X-100, 0.5% sodium deoxycholate, 0.1% SDS, 1× Halt protease inhibitor cocktail and 25 U ml^–1^ benzonase) by incubating on ice for at least 15 min. The lysates were cleared through centrifugation (15 min, 20,000*g*, 4 °C), and the total protein concentration was determined by BCA protein assay (Pierce BCA Protein Assay kit, Thermo Scientific, 23225) following the manufacturer’s protocol. All lysates were supplemented with 4× Bolt LDS sample buffer (Invitrogen) and denatured for 5 min at 95 °C before loading onto polyacrylamide gels (20 μg of total protein). Proteins were separated on 4–12% SDS–PAGE gels (Invitrogen) and transferred to nitrocellulose membranes. The transfer efficiency was tested through staining with Ponceau-S. The membranes were blocked with 5% milk in Tris-buffered saline with Tween20 (TBST) (30 min, room temperature). Primary antibodies were incubated overnight at 4 °C in 1% milk in TBST, while the respective secondary antibodies were incubated in TBST for 1 h at room temperature. Blots were developed with chemiluminescence films. The following primary antibodies were used: OSBP (1:2,000; Bethyl, A304-553A), GAPDH (1:5,000; Santa Cruz Biotechnology, sc-365062), GOLIM4 (1:1,000; Thermo Scientific, PA5-51624), PITPNB (1:2,000; Bethyl, A305-591A), GALNT2 (1:1,000; Abcam, ab262868), phospho-AKT (1:2,000; Cell Signaling Technology, 4060T), AKT (1:1,000; Cell Signaling Technology, 4691T) and HA (1:5,000; Cell Signaling Technology, 3724S). The following secondary antibody was used: peroxidase-conjugated AffiniPure goat anti-rabbit IgG (1:10,000; Jackson ImmunoResearch, 111-035-003).

### Expression proteomics

#### Sample preparation

Wild-type KBM7 cells (40 million) were treated with orpinolide (1 μM) or DMSO for 8 h in biological duplicates. Afterward, cells were collected via centrifugation, and the cell pellets were washed four times with ice-cold PBS before being flash-frozen in liquid nitrogen. Each sample was lysed in 500 μl of lysis buffer (50 mM HEPES (pH 8.0), 2% SDS, 1 mM PMSF and 1× protease inhibitor cocktail (Sigma-Aldrich)) for 20 min at room temperature before heating to 99 °C for 5 min. After cooling down to room temperature, the DNA was sheared by sonication (Covaris S2 high-performance ultrasonicator), and the lysates were cleared through centrifugation (20 min, 16,000*g*, 20 °C). Total protein concentration was determined by BCA protein assay (Pierce BCA Protein Assay kit, Thermo Scientific). Filter-aided sample preparation was performed using a 30-kDa molecular-weight-cutoff filter (VIVACON 500; Sartorius Stedim Biotech). In brief, 200 μg of total protein per sample was reduced with DTT (83.3 mM final concentration), followed by a 5-min incubation at 99 °C. After cooling to room temperature, samples were mixed with 200 μl of freshly prepared UA solution (8 M urea in 100 mM Tris-HCl (pH 8.5)), and the filter units were centrifuged (15 min, 14,000*g*, 20 °C) to remove SDS. Residual SDS was washed out by a second washing step with 200 μl of UA. The proteins were alkylated with 100 μl of 50 mM iodoacetamide for 30 min at room temperature in the absence of light. Afterward, three washing steps with 100 μl of UA solution were performed, followed by three washing steps with 100 μl of 50 mM TEAB buffer. Proteins were digested with trypsin at a ratio of 1:50 overnight at 37 °C. Peptides were recovered using 40 μl of 50 mM TEAB buffer, followed by 50 μl of 0.5 M NaCl and were desalted using C18 solid-phase extraction spin columns (The Nest Group). After desalting, peptides were labeled with TMT 16-plex reagents according to manufacturer’s protocol (Thermo Scientific Pierce). After quenching, all samples were pooled, organic solvent was concentrated under vacuum, and labeled peptides were cleaned via C18 solid-phase extraction.

#### Offline fractionation via reversed-phase high-performance liquid chromatography at high pH

Tryptic peptides were rebuffered in 10 mM ammonium formate (pH 10) before separation by reversed-phase (RP) liquid chromatography (LC) as previously described^71^. Peptides were separated into 96 time-based fractions on a Phenomenex C18 RP column (150 × 2.0 mm, Gemini-NX 3 μm C18 110 Å; Phenomenex) using a Dionex UltiMate 3000 RSLCnano system fitted with a binary high-pressure gradient pump delivering solvent at 50 µl min^–1^. Acidified fractions were consolidated into 36 fractions via a previously described concatenated strategy^72^. After removal of solvent in a vacuum concentrator, samples were reconstituted in 0.1% trifluoroacetic acid (TFA) before LC–tandem mass spectrometry (LC–MS/MS) analysis.

#### Data acquisition

LC–MS/MS analysis was performed on an Orbitrap Fusion Lumos Tribrid mass spectrometer (Thermo Scientific) coupled to a Dionex UltiMate 3000 RSLCnano system (Thermo Scientific) via a Nanospray Flex Ion Source (Thermo Scientific) interface. Peptides were loaded onto a trap column (PepMap 100 C18, 5 μm, 5 × 0.3 mm, Thermo Scientific) at a flow rate of 10 μl min^–1^ using 0.1% TFA as loading buffer. After loading, the trap column was switched in-line with a 75 μm (inner diameter) × 400 mm analytical column (packed in-house with ReproSil-Pur 120 C18-AQ, 3-μm particle size, Dr. Maisch HPLC). Mobile phase A consisted of 0.4% formic acid in water, while mobile phase B consisted of 0.4% formic acid in a mixture of 90% acetonitrile and 9.6% water. Separation was achieved using a multistep gradient over 90 min at a flow rate of 230 nl min^–1^ (increase of initial gradient from 6% to 9% solvent B within 1 min, 9% to 30 % solvent B within 81 min, 30% to 65% solvent B within 8 min, 65% to 100% solvent B within 1 min and 100% solvent B for 6 min before equilibrating to 6% solvent B for 18 min before the next injection). In the liquid junction setup, electrospray ionization was enabled by applying a voltage of 1.8 kV directly to the liquid being sprayed, and noncoated silica emitter was used. The mass spectrometer was operated in data-dependent acquisition mode and used a synchronous precursor selection (SPS) approach. For both MS^2^ and MS^3^ levels, we collected a survey scan from 400 to 1,600 *m*/*z* in the Orbitrap at 120,000 resolution (FTMS1), the automatic gain control (AGC) target was set to ‘standard’, and a maximum injection time (IT) of 50 ms was applied. Precursor ions were filtered by charge state (2–5), dynamic exclusion (120 s with a window of ±10 ppm) and monoisotopic precursor selection. Precursor ions for data-dependent MS^*n*^ analysis were selected using ten dependent scans (TopN approach). A charge state filter was used to select precursors for data-dependent scanning. In data-dependent MS^2^ analysis, spectra were obtained using one charge state per branch (from *z* = 2 to *z* = 5) in a dual-pressure linear ion trap (ITMS2). The quadrupole isolation window was set to 0.7 Da, and the collision-induced dissociation fragmentation technique was used at a normalized collision energy of 34%. The normalized AGC target was set to standard with a maximum IT of 35 ms. During the data-dependent MS^3^ analyses, precursors were isolated using SPS waveform and different MS^1^ isolation windows (1.3 *m*/*z* for *z* = 2, 1.2 *m*/*z* for *z* = 3, 0.8 *m*/*z* for *z* = 4 and 0.7 *m*/*z* for *z* = 5). Target MS^2^ fragment ions were further fragmented by high-energy collision-induced dissociation (HCD), followed by orbitrap analysis (FTMS3). The normalized HCD collision energy was set to 45%, and the normalized AGC target was set to 300% with a maximum IT set to ‘auto’. The resolution was set to 50,000 with a defined scanning range of 100 to 500 *m*/*z*. Xcalibur (v.3.3.2782.34) and Tune (v.3.3) were used to operate the instrument.

#### Data analysis

Acquired raw data files were processed with Proteome Discoverer (v.2.4.1.15) using the Sequest HT database search engine and Percolator validation software node to remove false positives with an FDR of 1% on peptide and protein levels under strict conditions. Searches were performed with full tryptic digestion against the human SwissProt database (*Homo sapiens*; SwissProt TaxID 9606; v2017; 42,252 sequences) with or without deamidation (+0.9840 Da) on amino acids aspartic acid and glutamic acid. Methionine oxidation (+15.994 Da) and protein N-terminal acetylation (+42.011 Da) as well as methionine loss (–131.040 Da) and protein N-terminal acetylation with methionine loss (–89.030 Da) were set as variable modifications, while carbamidomethylation (+57.021 Da) of cysteine residues and TMT 16-plex labeling of peptide N-termini and lysine residues (+304.207 Da) were set as fixed modifications. Data were searched with mass tolerances of ±10 ppm and ±0.6 Da on the precursor and fragment ions, respectively. Results were filtered to include peptide spectrum matches (PSMs) with Sequest HT cross-correlation factor scores of ≥1 and high peptide confidence assigned by Percolator. MS^3^ signal-to-noise values of TMT reporter ions were used to estimate peptide/protein abundance changes. PSMs with precursor isolation interference values of ≥70%, SPS mass matches of ≤65% and average TMT reporter ion signal-to-noise values of ≤10 were excluded from quantitation. Both unique and razor peptides were used for TMT quantitation. Isotopic impurity correction and TMT channel normalization based on total peptide amount were applied. For statistical analysis and *P* value calculation, the integrated ANOVA hypothesis test was used. TMT ratios with *P* values below 0.05 were considered significant. Only proteins with more than one peptide detected and more than one protein unique peptide detected were considered for further analysis. GO enrichment analysis of downregulated proteins was performed using tools available on the GO Consortium website (http://geneontology.org; release 1 January 2023).

### Protein interaction analysis of up- and downregulated proteins

A full proteome profiling, using the TMT technique, of orpinolide- versus DMSO-treated cells identified 59 up- and 116 downregulated proteins (175 regulated proteins; log_2_-transformed fold change greater than 0.25 or less than –0.25, adjusted *P* value of <0.05). Localization of each protein was annotated by combining the localization information from The Human Protein Atlas project (v.22.0)^73^ and UniProtKB (13.01.2023). The different localization information was further manually curated, considering the multiple localizations and corresponding confidence levels. From the interaction database BioGRID (v.4.4.212)^21^, a PPI network limited to direct physical interactions between the regulated proteins was derived. For this, first, the BioGRID database was filtered to only include interactions reported for *H. sapiens* (9606). Further, only interactions that were annotated to be generated by ‘affinity capture-western’, ‘co-purification’ and ‘affinity capture-MS’ were used, omitting all proximity interaction and coelution profiling technologies. This resulted in an interaction database with 610,485 interactions. The filtered BioGRID database contained nodes for 172 of the 175 regulated proteins. Next, all edges between the regulated proteins were extracted, resulting in a network covering 97 nodes (55% of all regulated proteins) and 97 edges connecting the nodes. Seventy-five regulated proteins were not directly connected to other reported regulated proteins. We sampled 172 random nodes 10,000 times from the filtered BioGRID database and obtained a higher number of nodes (permutation *P* = 0.0097) for the reconstructed network of regulated proteins, while none of the permutated networks had a higher or equal number of edges (permutation *P* = 0), indicating that the up- and downregulated proteins represent a significantly more than random related set of proteins. Data analysis was conducted with the statistical software R (version R-4.2.0), while the network was visualized using the software Cytoscape (v.3.8.0).

### RNA sequencing

Wild-type KBM7 cells (10 million) were treated with orpinolide (485 nM) or DMSO for 6 h in biological triplicates. Total RNA was extracted with an RNeasy Mini kit (Qiagen, 74106), and RNA quantity was determined using a Qubit RNA HS kit (Thermo, Q32852). Next, poly(A) enrichment (Lexogen, 039) was performed with 4 µg of RNA per condition, and the RNA-sequencing library was prepped using a Corall Total RNA-Seq Library Prep kit (Lexogen, 095) following the manufacturer’s protocol (2 ng of RNA as starting material). End point PCR was performed with 16 cycles. Amplified and purified sequencing libraries were analyzed on an Agilent 2100 Bioanalyzer (High-Sensitivity DNA Analysis kit, Agilent, 5067-4626), pooled in equimolar amounts (2.47 ng μl^–1^) and sequenced using an Illumina HiSeq 4000 platform and 50-base pair (bp) single-end configuration.

#### RNA-sequencing data analysis

Raw reads were trimmed using Trimmomatic (v.0.32)^74^. The following parameters were used: HEADCROP:13, ILLUMINACLIP:epignome_adapters_2_add.fa:2:10:4:1:true, SLIDINGWINDOW:4:1, MAXINFO:16:0.40 and MINLEN:30. Trimmed reads were mapped to the hg38/GRCh38 assembly of the human reference genome using STAR aligner (v.2.5.2b)^75^ using the following parameters: –outFilterType BySJout–outFilterMultimapNmax 20–alignSJoverhangMin 8–alignSJDBoverhangMin 1–outFilterMismatchNmax 999–outFilterMismatchNoverLmax 0.6–alignIntronMin 20–alignIntronMax 1000000–alignMatesGapMax 1000000–outSAMattributes NH HI NM MD–outSAMtype BAM SortedByCoordinate. Genes were defined based on genome build GRCh38.p7, genome build accession NCBI GCA_000001405.22. Read counts per gene were obtained from the aligned reads using the htseq-count command (v.0.11.2) from the HTSeq framework^76^. The Bioconductor/R package DESeq2 (v.1.34.0)^77^ was used for normalization and differential gene expression analysis. A GSEA was performed using GSEA software (v.3.0)^78^, and the results were represented using R. To build gene rankings, genes were ranked based on metascore values. The metascore represents ‘the worst’ score from the following three scores obtained from the DESeq2 analysis: (1) log_2_-transformed fold change, (2) baseMean RNA-sequencing score and (3) *P* value. The following GSEA parameters were used: xtools.gsea.GseaPreranked -nperm 1000 -scoring_scheme weighted -norm meandiv.

### Genome-wide CRISPR–Cas9 knockout screen

Genome-wide CRISPR–Cas9 positive selection screens were performed as previously reported^62^. KBM7-Cas9 cells (250 million) were transduced with the Brunello sgRNA library (Addgene, 12260) at a multiplicity of infection of 0.23 to yield a calculated library representation of 758 cells per sgRNA. Transductions were performed as described above. The next day, the transduced cells were pooled and diluted with fresh IMDM. Pools were selected with puromycin (1 μg ml^–1^) for 5 days, after which, drug treatments were started. Selected pools (50 million cells; density of 0.5 million cells per ml) were treated with DMSO or orpinolide (243 nM starting concentration). Every 4 days, cells were counted, and 50 million cells were reseeded (maintaining a density of 0.5 million cells per ml) and treated with fresh orpinolide or DMSO. Orpinolide concentration was dynamically adjusted to the accumulated growth curves to maintain a consistent impact on cell proliferation without losing coverage. After 20 days of treatment, lymphocyte separation medium (Corning) was used to remove dead cells and cellular debris. Viable cells were collected, and the cell pellets were flash-frozen in liquid nitrogen.

#### Library preparation for next-generation sequencing

Genomic DNA was isolated from respective samples with a QIAamp DNA Mini kit (Qiagen, 51306) and used as template for PCR amplification of sgRNA sequences in batches of 20 μg of genomic DNA per PCR reaction. One PCR reaction (100 μl) furthermore contained P5 forward primer mix (0.5 μM), condition-specific P7 barcoded primer (0.5 μM), ExTaq polymerase (Clontech, 1.5 μl), dNTP mix (8 μl), 10× buffer (10 μl) and water. The primer sequences used are listed in Supplementary Table 8. The following parameters were used for target amplification: 1 min at 95 °C initial denaturation; 30 s at 95 °C, 30 s at 53 °C and 30 s at 72 °C for 27 cycles and 10 min at 72 °C final elongation. After pooling all PCR reactions for a specific condition, the 360-bp amplicon was purified with AMPure XP beads (Beckman Coulter, 10136224; 0.7× right side size selection) and eluted with TE buffer (28 μl). Sequencing libraries were analyzed on an Agilent 2100 Bioanalyzer (High-Sensitivity DNA Analysis kit, Agilent, 5067-4626) pooled in equimolar amounts (2.8 ng μl^–1^) and sequenced using an Illumina HiSeq 4000 platform and 50-bp single-end configuration.

#### Next-generation sequencing data analysis

Raw read files were converted to fastq format using the convert function from BamTools (v.2.5.2)^79^. Sequencing adapters were trimmed using Cutadapt (v.3.4)^80^ with -g CGAAACACCG and –minimum-length = 10. The 20 bp of spacer sequence was then extracted using the fastx toolkit (v.0.0.14; http://hannonlab.cshl.edu/fastx_toolkit/) and aligned to the respective sgRNA index using Bowtie2 (v.2.4.4)^81^, allowing for one mismatch in the seed sequence. Spacers were counted using the bash command ‘cut -f 3 (0) | sort | uniq -c’ on the sorted SAM files. A count table with all conditions was then assembled, and the counts + 1 were converted to counts per million to normalize for sequencing depth. The log_2_-normalized fold change values compared to DMSO were calculated for each spacer. Statistical analysis was performed using the STARS algorithm (v.1.3)^25^. For this, spacers were rank ordered based on log_2_-transformed fold change and tested with the parameters –thr 10–dir P against a null hypothesis of 1,000 random permutations. Genes with an FDR *P* value of <0.05 were called hits.

### Thermal proteome profiling in intact cells

#### Sample preparation

KBM7 cells (36 million, density of 1.5 million cells per ml) were treated with orpinolide (5 μM) or DMSO for 2 h in biological duplicates. Afterward, cells were collected via centrifugation, washed three times with ice-cold PBS and transferred into PCR plates so that each well contained 4 million cells (100 μl). After spinning down, 80 μl of the supernatant was removed without disturbing the pellet. The samples were then thermally destabilized (3 min at an eight-temperature gradient (37.3, 42.7, 46.2, 49.4, 52.7, 55.9, 59.4 and 62.3 °C), followed by 3 min at 25 °C) and resuspended in 60 μl of lysis buffer (20 mM Tris-HCl (pH 8.0), 120 mM NaCl, 0.5% NP-40 and 1× Halt protease inhibitor cocktail). Four freeze/thaw cycles with liquid nitrogen were then performed, and the lysed samples were cleared via centrifugation (20,817*g*, 1 h, 4 °C). In total, 38 lysates were obtained, with each biological replicate comprising 16 DMSO/orpinolide-treated samples destabilized at eight different temperatures. Cleared supernatants (70 μl) were supplemented with SDS to a final concentration of 2%, and the protein concentration was determined by BCA protein assay (Pierce BCA Protein Assay kit, Thermo Scientific). Filter-aided sample preparation was performed, as described in Expression proteomics, using a 30-kDa molecular-weight-cutoff filter. After reduction and alkylation, proteins (in 50 μl of 50 mM TEAB buffer) were digested with trypsin (3 μg per sample; the protein input for digest was between 40 and 150 μg depending on the temperature condition) overnight at 37 °C, followed by the addition of fresh trypsin (4.5 μg per sample) for an additional 3 h. Peptides were recovered using 40 μl of 50 mM TEAB buffer followed by 50 μl of 0.5 M NaCl and were desalted using Pierce Peptide Desalting Spin Columns (Thermo Scientific). Desalted peptides were subsequently labeled with TMT 16-plex reagents (two 16-plexes in total, one for each biological replicate) according to manufacturer’s protocol (Thermo Scientific Pierce). One TMT 16-plex comprised eight temperature points of orpinolide and eight temperature points of DMSO-treated cells. After quenching, all samples per respective 16-plex were pooled, organic solvent was concentrated under vacuum, and labeled peptides were cleaned via C18 solid-phase extraction.

#### Offline fractionation and data acquisition

Offline fractionation via RP high-performance LC at high pH (20 fractions per 16-plex) was performed as described in Expression proteomics.

#### Data acquisition

LC–MS/MS analysis was performed on an Orbitrap Fusion Lumos Tribrid mass spectrometer (Thermo Scientific) coupled to a Dionex UltiMate 3000 RSLCnano system (Thermo Scientific) via a Nanospray Flex Ion Source (Thermo Scientific) interface. Peptides were loaded onto a trap column (PepMap 100 C18, 5 μm, 5 × 0.3 mm, Thermo Scientific) at a flow rate of 10 μl min^–1^ using 0.1% TFA as loading buffer. After loading, the trap column was switched in-line with an Acclaim PepMap nanoHPLC C18 analytical column (2.0-μm particle size, 75 μm (inner diameter) × 500 mm; 164942, Thermo Scientific). The column temperature was maintained at 50 °C. Mobile phase A consisted of 0.4% formic acid in water, while mobile phase B consisted of 0.4% formic acid in a mixture of 90% acetonitrile and 9.6% water. Separation was achieved using a multistep gradient over 90 min at a flow rate of 230 nl min^–1^ (increase of initial gradient from 6% to 9% solvent B within 1 min, 9% to 30% solvent B within 81 min, 30% to 65% solvent B within 8 min, 65% to 100% solvent B within 1 min and 100% solvent B for 6 min before equilibrating to 6% solvent B for 18 min before the next injection). In the liquid junction setup, electrospray ionization was enabled by applying a voltage of 1.8 kV directly to the liquid being sprayed, and noncoated silica emitter was used. The mass spectrometer was operated in data-dependent acquisition mode and used an SPS approach. For both MS^2^ and MS^3^ levels, we collected a survey scan from 400 to 1,650 *m*/*z* in the Orbitrap at 120,000 resolution (FTMS1), the AGC target was set to ‘standard’, and a maximum IT of 50 ms was applied. Precursor ions were filtered by charge state (2–6), dynamic exclusion (60 s with a window of ±10 ppm) and monoisotopic precursor selection. Precursor ions for data-dependent MS^*n*^ analysis were selected using ten dependent scans (TopN approach). A charge state filter was used to select precursors for data-dependent scanning. In data-dependent MS^2^ analysis, spectra were obtained using one charge state per branch (from *z* = 2 to *z* = 5) in a dual-pressure linear ion trap (ITMS2). The quadrupole isolation window was set to 0.7 Da, and the collision-induced dissociation fragmentation technique was used at a normalized collision energy of 35%. The normalized AGC target was set to 200% with a maximum IT of 35 ms. During the data-dependent MS^3^ analyses, precursors were isolated using SPS waveform and different MS^1^ isolation windows (1.3 *m*/*z* for *z* = 2, 1.2 *m*/*z* for *z* = 3, 0.8 *m*/*z* for *z* = 4 and 0.7 *m*/*z* for *z* = 5). Target MS^2^ fragment ions were further fragmented by HCD, followed by orbitrap analysis (FTMS3). The normalized HCD collision energy was set to 45%, and the normalized AGC target was set to 300% with a maximum IT set to ‘auto’. The resolution was set to 50,000 with a defined scanning range of 100 to 500 *m*/*z*. Xcalibur (v.4.3.73.11) and Tune (v.3.4.3072.18) were used to operate the instrument.

#### Data analysis

First, common lab contaminants were filtered from the datasets and the abundance values of the biological replicates. Next, we normalized all measurements (*n* = 4) per temperature point by applying a sum total normalization (see Supplementary Fig. 2). Only proteins quantified greater than or equal to two PSMs across the DMSO- (vehicle) and compound-treated conditions were considered (removal of 470 ProteinGroups across all conditions). Next, the relative abundance of each protein per condition and replicate was scaled to 37 °C, the lowest temperature point. Subsequently, we used the NPARC R package (v.1.2.0)^34^ to identify significant shifts in protein melting behavior after compound treatment. The NPARC method has the advantage of identifying subtle but highly reproducible *T*_m_ shifts by comparing protein melting curves, including goodness of fit and residual errors of the model, and does not rely on the estimated *T*_m_ point. Before model fitting, the dataset was filtered to proteins quantified in both biological replicates. This resulted in 4,682 proteins (from 5,969 in total), which were used for the NPARC method. We obtained in vivo melting curves for 4,037 proteins, and 113 were found to show a significant shift in *T*_m_ with an adjusted *P* value below 0.01. The obtained hits were further filtered to yield proteins with a Δ*T*_m_ exceeding 1 °C (see Supplementary Table 5).

### Cellular thermal shift assay in intact cells

After treatment with orpinolide or DMSO (1 million cells per ml), wild-type KBM7 cells or KBM7 cells overexpressing HiBiT-tagged proteins of interest were spun down (1,450 rpm, 5 min), washed once with PBS and resuspended in PBS (100 μl of PBS per 1 million cells). The cell suspension (100 μl) was distributed into PCR strips and spun down (1,450 rpm, 5 min), and 80 μl of the supernatant was removed without disturbing the cell pellet. The samples were then thermally destabilized (3-min temperature gradient of choice followed by 3 min at 25 °C) and resuspended in 30 μl of lysis buffer (20 mM Tris-HCl (pH 8.0), 120 mM NaCl and 0.5% NP-40). Three freeze/thaw cycles with liquid nitrogen were then performed, and the lysed samples were cleared out via centrifugation (full speed, 20 min, 4 °C).

#### Analysis via immunoblotting

Cleared supernatants were supplemented with 4× Bolt LDS sample buffer (Invitrogen) and denatured for 5 min at 95 °C before loading (6 μl) onto 4–12% polyacrylamide gels. Western blotting was performed as described above.

#### HiBiT cellular thermal shift assay

Luminescence was assessed with a Nano-Glo HiBiT Lytic Detection System (Promega, N3040) in 384-well plate format and in technical triplicates. Briefly, a 6× detection master mix was prepared containing the substrate, LgBiT and the provided lysis buffer, and 2.5 μl was distributed into well plates. Cleared lysates of cells expressing HiBiT-tagged proteins of interest (12.5 μl) were then added into respective wells, and the plates were incubated at room temperature for 10 min with mild shaking. Luminescence signal was measured on a Multilabel Plate Reader Platform Victor X3 model 2030 (PerkinElmer). Melting curves and *T*_m_ were determined in GraphPad Prism (v.9.5.1) by fitting a nonlinear regression curve or interpolation of a sigmoidal standard curve. Each data point was normalized to the mean luminescence at the lowest temperature (typically 37.6 °C).

### Immunofluorescence and confocal microscopy

Cells were grown to 70% confluency on glass coverslips and fixed in 4% paraformaldehyde in PBS for 10 min at room temperature. Fixed cells were blocked and permeabilized for 20 min in blocking buffer (0.05% (wt/vol) saponin, 0.5% (wt/vol) bovine serum albumin, 50 mM NH_4_Cl and 0.02% NaN_3_ in PBS, pH 7.4). Cells were stained with primary antibodies diluted in blocking buffer for 1 h at room temperature. The following primary antibodies were used: OSBP (1:150; Atlas Antibodies, HOA039227), TGN46 (1:200; Bio-Rad, AHP500GT), PC2 (recognizes folded PC2 only; 1:200; Hybridoma Bank, II6B3) and giantin (1:100; Institut Curie, recombinant proteins platform, A-R-R#05). After three washes with PBS, cells were stained with Alexa Fluor 488-, 568- and 647-conjugated secondary antibodies (Invitrogen, goat anti-rabbit 488 (A11008), goat anti-mouse 488 (A11001), goat anti-mouse 568 (A11031) and donkey anti-sheep 647 (A21448)) diluted in blocking buffer (1:400) for 45 min at room temperature. Cells were washed three times with PBS, and the nuclei were counterstained for 5 min with Hoechst 33342 (Invitrogen) before being mounted on glass coverslips using Fluoromount-G (Invitrogen). Cell images were acquired on an SP5 scanning confocal (Leica) with a ×63/1.4-NA Plan Apo oil objective lens (Zeiss) with a pixel size of 0.161 μm and step size of 0.4 μm. Images were processed and quantified using Fiji ImageJ2 (National Institute of Health)^82^.

#### Golgi fragmentation assay

HeLa cells were treated with 100 nM or 1 μM orpinolide or vehicle (DMSO) for 4 h or 8 h at 37 °C before fixation and immunofluorescence. Quantification of Golgi fragmentation was semiautomated by applying Max Entropy automatic threshold to individual cells to determine areas of positive fluorescence (for TGN46) and counting the number of fragments above threshold per cell by using the ‘analyze particles’ command. For quantifying OSBP localization, Integrated Density of OSBP was measured in the Golgi region of interest (TGN46 as defined above) and normalized to Integrated Density for OSPB in the whole cell.

#### Anterograde trafficking assay

RCS cells treated with 1 μM orpinolide or vehicle (DMSO) were incubated in a water bath at 40 °C for 4 h in DMEM containing 10% FBS and 20 nM HEPES (pH 7.2). Cycloheximide (100 μg ml^–1^) and ascorbic acid (50 μg ml^–1^) were added to the trafficking medium, and cells were switched to a water bath set at 32 °C. Subsequently, cells were fixed with 4% paraformaldehyde at the specified time points (0–45 min), and immunofluorescence staining was conducted. Quantification of PC2 trafficking was semiautomated by applying Max Entropy automatic threshold to individual cells to determine areas of positive fluorescence for giantin to make the Golgi mask. Integrated Density of PC2 in the Golgi region of interest was measured and normalized to Integrated Density of PC2 in the whole cell.

#### Statistics

Statistical analyses were performed using GraphPad Prism (v.9.5.1). In cases where the data did not exhibit a normal distribution, log_10_ transformation was applied to normalize the distribution. A one-way ANOVA (Golgi fragmentation) or two-way ANOVA (anterograde trafficking assay) was applied with a Dunnett’s or Šídák’s multiple comparison test to compare each treatment group to the DMSO control. A *P* value of 0.05 or less was considered statistically significant. The number of cells quantified from independent experiments is specified in the figure legends.

### Recombinant proteins

#### Protein expression and purification

ASTER domains of human Aster-A_359–547_, Aster-B_364–552_ and Aster-C_318–504_ in the pGEX-6p-2rps vector with an N-terminal PreScission-cleavable GST tag were expressed in *Escherichia coli* OverExpress C41 in Terrific Broth for approximately 16 h at 18 °C after induction with 0.1 mM isopropyl β-d-1-thiogalactopyranoside (IPTG). Cells were collected at 3,500*g* for 15 min and lysed by sonication in buffer containing 50 mM HEPES (pH 7.5), 300 mM NaCl, 10% (vol/vol) glycerol, 5 mM DTT, 0.1% (vol/vol) Triton X-100 and protease inhibitor mix HP plus (Serva). The cleared lysate was purified by affinity chromatography on a GSTrap FF column (Cytiva) using an ÄKTA Start (Cytiva) in buffer containing 50 mM HEPES (pH 7.5), 300 mM NaCl, 10% (vol/vol) glycerol, 5 mM DTT and 0.01% (vol/vol) Triton X-100. The GST tag was cleaved on the column overnight at 4 °C. Proteins were further purified by size-exclusion chromatography on a HiLoad 16/600 Superdex 75 pg (Cytiva) in buffer containing 20 mM HEPES (pH 7.5), 300 mM NaCl, 10% (vol/vol) glycerol and 2 mM DTT.

The START domain of human STARD1_66–284_ harboring an N-terminal His_6_ tag was expressed in *E. coli* BL21(DE3) in Luria–Bertani broth for approximately 16 h at 18 °C after induction with 0.15 mM IPTG. Cells were collected at 3,500*g* for 15 min and lysed by sonication in buffer containing 50 mM HEPES (pH 7.5), 150 mM NaCl, 5% (vol/vol) glycerol, 5 mM DTT, 0.1% (vol/vol) Triton X-100 and EDTA-free protease inhibitor cocktail (Sigma-Aldrich). The cleared lysate was purified by affinity chromatography on an Ni-NTA Superflow Cartridge (Qiagen) using an ÄKTA Start (Cytiva) in buffer containing 50 mM HEPES (pH 7.5), 150 mM NaCl, 5% (vol/vol) glycerol and 5 mM DTT. STARD1_66–284_ was eluted with buffer containing 50 mM HEPES (pH 7.5), 150 mM NaCl, 5% (vol/vol) glycerol, 5 mM DTT and 500 mM imidazole. Proteins were further purified by size-exclusion chromatography on a HiLoad 16/600 Superdex 75 pg (Cytiva) in buffer containing 20 mM HEPES (pH 7.5), 150 mM NaCl, 5% (vol/vol) glycerol and 2 mM DTT.

The ORP domain of human OSBP_377–807_ in the pGEX-6p-1 vector with an N-terminal PreScission-cleavable GST tag was expressed in *E. coli* OverExpress C41 in Luria–Bertani broth for approximately 16 h at 18 °C after induction with 0.1 mM IPTG. Cells were collected at 3,500*g* for 15 min and lysed by sonication in buffer containing 20 mM HEPES (pH 7.5), 300 mM NaCl, 10% (vol/vol) glycerol, 5 mM DTT, 0.1% (vol/vol) Triton X-100 and EDTA-free protease inhibitor cocktail (Sigma-Aldrich). The cleared lysate was purified by affinity chromatography on a GSTrap HF column (Cytiva) using an ÄKTA Start (Cytiva) in buffer containing 20 mM HEPES (pH 7.5), 300 mM NaCl, 10% (vol/vol) glycerol and 5 mM DTT. OSBP_377–807_ was eluted with buffer containing 20 mM HEPES (pH 7.5), 300 mM NaCl, 10% (vol/vol) glycerol, 5 mM DTT and 10 mM reduced glutathione. Proteins were further purified by size-exclusion chromatography on a HiLoad 16/600 Superdex 75 pg (Cytiva) using an ÄKTA Explorer (Cytiva) in buffer containing 20 mM HEPES (pH 7.5), 150 mM NaCl, 10% (vol/vol) glycerol and 2 mM DTT.

### Fluorescence polarization

Fluorescence polarization experiments were performed in a buffer composed of 20 mM HEPES (pH 7.5), 300 mM NaCl, 0.01% (vol/vol) Tween 20, 0.5% glycerol and 2 mM DTT in a final volume of 30 μl in black, flat-bottom, nonbinding 384-well plates (Corning). For competition experiments, 20 nM 22-NBD-cholesterol was mixed with protein and incubated with the desired concentrations of screening compounds. The fluorescence polarization signal was measured using a Spark Cyto multimode microplate reader (Tecan) with filters set at 485 ± 20 nm for excitation and at 535 ± 20 nm for emission. The data were analyzed using GraphPad Prism (v.9.5.1). Measured millipolarization values were normalized setting 100% inhibition as the fluorescence polarization signal from the protein + fluorophore control well and 0% as the fluorescence polarization signal from the fluorophore control well. Curves were fitted to the normalized data via nonlinear regression to allow the determination of half-maximal inhibitory concentration values.

### Sterol transfer assay

#### Vesicle preparation

Stock solutions of the required lipids were prepared as follows: 1,2-dioleoyl-*sn*-glycero-3-phosphocholine (DOPC; Avanti Polar Lipids, 850375C) in chloroform (10 mg ml^–1^) and 23-(dipyrrometheneboron difluoride)-24-norcholesterol (TopFluor Cholesterol, Avanti Polar Lipids, 810255) and *N*-(lissamine rhodamine B sulfonyl)-1,2-dihexadecanoyl-*sn*-glycero-3-phosphoethanolamine (triethylammonium salt) (Rh-DHPE; Invitrogen, L1392) in methanol (100 μM).

L_D_ were prepared by mixing the DOPC–TF-Chol–Rh-DHPE stock solutions in a molar ratio of 99:0.5:0.5, and the L_A_ contained only DOPC (final volume of 1 ml). Evaporation of the solvent under a stream of nitrogen, followed by drying under vacuum overnight, afforded the dried lipid films. The lipid films were hydrated to a final concentration of 60 μM in a buffer containing 20 mM HEPES (pH 7.5), 300 mM NaCl and 2 mM DTT. Obtained solutions were extensively vortexed until full hydration was observed and sonicated for 5 min in a water bath at 40 °C, followed by five freeze/thaw cycles in liquid nitrogen. Homogenous unilamellar vesicles were obtained by extrusion through a polycarbonate membrane (21 times, 0.1-μm pore size; Avanti Polar Lipids) at 40 °C. Solutions were kept on ice and used on the day of preparation.

#### Microplate-based cholesterol transfer assay

The experiment was conducted using nonbinding, clear-bottom 96-well plates (Greiner Bio-One, 655906). The GST-tagged ORP domain of human OSBP_377–807_ was preincubated with either DMSO or orpinolide (10 mM DMSO stock solution) at room temperature for 30 min in buffer (20 mM HEPES (pH 7.5), 300 mM NaCl and 2 mM DTT), resulting in a final concentration of 500 nM for both the protein and the compound. Before measuring the FRET signal, equal amounts of L_A_ and L_D_ were added to the wells, resulting in a final concentration of 16 µM for both L_A_ and L_D_. After 2.5 min, GST–OSBP_377–807_ preincubated with either DMSO or orpinolide was added and rapidly mixed using a pipette before the measurement continued. Additional control wells included just the L_A_ and L_D_ mixture without added protein or compound. Fluorescence intensity measurements were performed in a Spark Cyto plate reader (Tecan) at 25 °C, with readings taken from the bottom of the wells at 10-s intervals for a duration of 15 min. The excitation monochromator was set at 488 ± 20 nm, and the emission monochromator was set at 590 ± 20 nm.

### Flow cytometry analysis of OSBP/ORP4 essentiality

To quantify the influence of OSBP and/or ORP4 genetic perturbation, KBM7 cells expressing iCas9 or Jurkat cells constitutively expressing Cas9 were transduced with pLenti-U6-sgRNA1-U6-sgRNA2-EF1αs-eBFP2 reporter with 50–60% infection efficiency. The reporter plasmid carries a combination of sgRNAs targeting the *OSBP*, *OSBP2* or *AAVS1* locus to yield single or dual *OSBP*/*OSBP2* knockouts (Supplementary Table 7). In KBM7 iCas9 cells, infection levels were determined by flow cytometry 3 days after transduction based on BFP marker expression (day 0), and Cas9 expression was induced with doxycycline (0.4 μg ml^–1^). In Jurkat-Cas9 cells, infection levels were determined by flow cytometry 3 days after transduction. In both cases, the percentage of sgRNA^+^ (BFP^+^) cells was monitored by flow cytometry in regular intervals. Flow cytometry measurements were performed on an LSRFortessa (BD Biosciences) using BD FACSDiva software (v.9.0), and the data were analyzed in FlowJo (v.10.8.1). For flow cytometry gating strategies, see Supplementary Fig. 3.

### Reporting summary

Further information on research design is available in the Nature Portfolio Reporting Summary linked to this article.

## Online content

Any methods, additional references, Nature Portfolio reporting summaries, source data, extended data, supplementary information, acknowledgements, peer review information; details of author contributions and competing interests; and statements of data and code availability are available at 10.1038/s41589-024-01614-4.

## Supplementary information

Supplementary Information Supplementary Tables 7 and 8 and Figs. 1–4.

Reporting Summary

Supplementary Table 1 Phenotypic profiling of the withanolide-inspired compound collection.

Supplementary Table 2 Expression proteomics.

Supplementary Table 3 RNA sequencing.

Supplementary Table 4 Genome-wide CRISPR–Cas9 resistance screen.

Supplementary Table 5 TPP.

Supplementary Table 6 Sequences of cDNAs and synthesized fragments.

Supplementary Data 1 Source data for Supplementary Figs. 1 and 2.

## Source data

Source Data Fig. 1 Statistical source data.

Source Data Fig. 2 Statistical source data.

Source Data Fig. 3 Statistical source data.

Source Data Fig. 4 Unprocessed western blots.

Source Data Fig. 4 Statistical source data.

Source Data Fig. 5 Unprocessed western blots.

Source Data Fig. 5 Statistical source data.

Source Data Extended Data Fig. 2 Statistical source data.

Source Data Extended Data Fig. 3 Statistical source data.

Source Data Extended Data Fig. 4 Statistical source data.

Source Data Extended Data Fig. 4 Unprocessed western blots.

Source Data Extended Data Fig. 5 Statistical source data.

Source Data Extended Data Fig. 5 Unprocessed western blots.

Source Data Extended Data Fig. 6 Statistical source data.

Source Data Extended Data Fig. 7 Statistical source data.

Source Data Extended Data Fig. 7 Unprocessed western blots.

Source Data Extended Data Fig. 8 Statistical source data.

Source Data Extended Data Fig. 9 Statistical source data.

Source Data Extended Data Fig. 10 Statistical source data.

Source Data Extended Data Fig. 10 Unprocessed western blots.

## Data Availability

The MS proteomics data (Figs. 2a,b and 4a, Extended Data Figs. 4 and 6 and Supplementary Tables 2 and 5) have been deposited to the ProteomeXchange Consortium via the PRIDE^83^ partner repository with the dataset identifiers PXD040694 and PXD040694 (expression proteomics) as well as PXD040692 and PXD040692 (TPP). Raw and analyzed RNA-sequencing and genome-wide CRISPR–Cas9 screening datasets (Figs. 2c,d and 3, Extended Data Fig. 5 and Supplementary Tables 3 and 4) are available in NCBI’s Gene Expression Omnibus under accession number GSE226849. Additionally, publicly available data from the following databases were used in this study: DepMap (22Q4 and 23Q2), The Human Protein Atlas project (v.22.0), UniProtKB (13.01.2023) and BioGRID (v.4.4.212). The data supporting all of the findings in this study are available within the paper, its supplementary files and the mentioned databases. Source data are provided with this paper.

## Code availability

The data analysis code is available at https://github.com/GWinterLab/W7.
