# Supplementary material for: Orpinolide disrupts a leukemic dependency on cholesterol transport by inhibiting OSBP
Source: Nat Chem Biol. 2024 Jun 21;21(2):193–202. doi: 10.1038/s41589-024-01614-4 (PMC11782089; doi:10.1038/s41589-024-01614-4)
Supplement: Supplementary file 1 — Supplementary Tables 7 and 8 and Figs. 1–4. [file 41589_2024_1614_MOESM1_ESM.pdf]

nature chemical biology

Supplementary information https://doi.org/10.1038/s41589-024-01614-4

Orpinolide disrupts a leukemic dependency on cholesterol transport by inhibiting OSBP

In the format provided by the authors and unedited

Table of contents

Supplementary Table 7. Plasmids and sgRNAs used in this study.
Supplementary Table 8. Primers used for PCR-amplification of sgRNAs in the genome-wide CRISPR/Cas9 screen.
Supplementary Figure 1. Thermal proteome profiling.
Supplementary Figure 2. Quantification of Golgi morphology alterations upon orpinolide treatment.
Supplementary Figure 3. Gating strategy used for flow cytometry experiments.
Supplementary Figure 4. Purity of orpinolide (W7) analyzed via LC-MS.

Supplementary Table 7. Plasmids and sgRNAs used in this study.

Plasmid name		Application
LentiGuide-Puro-sg*AAVS1*_1		CRISPR screen validation
LentiGuide-Puro-sg*AAVS1*_2		CRISPR screen validation
LentiGuide-Puro-sg*ARMH3*_1		CRISPR screen validation
LentiGuide-Puro-sg*ARMH3*_2		CRISPR screen validation
LentiGuide-Puro-sg*ARMH3*_3		CRISPR screen validation
LentiGuide-Puro-sg*PITPNB*_1		CRISPR screen validation
LentiGuide-Puro-sg*PITPNB*_2		CRISPR screen validation
LentiGuide-Puro-sg*PITPNB*_3		CRISPR screen validation
pLEX306-OSBP-V5-HiBiT		HiBiT-tagged fusion
pLEX306-ORP1-HiBiT		HiBiT-tagged fusion
pLEX306-ORP2-V5-HiBiT		HiBiT-tagged fusion
pLEX306-ORP4L-V5-HiBiT		HiBiT-tagged fusion
pLEX306-ORP9-V5-HiBiT		HiBiT-tagged fusion
pLEX306-ORP11-V5-HiBiT		HiBiT-tagged fusion
pLEX306-RAB33A-HiBiT		HiBiT-tagged fusion
pLEX306-TM9SF4-HiBiT		HiBiT-tagged fusion
pLEX-OSBP$^{wt/M446W}$-V5-2HA-P2A-Puro		HA-tagged wt/mutant fusion
pLenti-U6-sg*AAVS1*_1-U6-sg*AAVS1*_2-EF1αs-eBFP2		Dropout screen
pLenti-U6-sg*OSBP*_1-U6-sg*AAVS1*_1-EF1αs-eBFP2		Dropout screen
pLenti-U6-sg*OSBP*_2-U6-sg*AAVS1*_1-EF1αs-eBFP2		Dropout screen
pLenti-U6-sg*AAVS1*_1-U6-sg*OSBP2*_1-EF1αs-eBFP2		Dropout screen
pLenti-U6-sg*AAVS1*_1-U6-sg*OSBP2*_2-EF1αs-eBFP2		Dropout screen
pLenti-U6-sg*OSBP*_1-U6-sg*OSBP2*_1-EF1αs-eBFP2		Dropout screen
pLenti-U6-sg*OSBP*_2-U6-sg*OSBP2*_2-EF1αs-eBFP2		Dropout screen
pGEX-6p-1-GST-OSBP$_{377-807}$		Recombinant expression
pGEX-6p-2rbs-Aster-A$_{359-547}$		Recombinant expression
pGEX-6p-2rbs-Aster-B$_{364-552}$		Recombinant expression
pGEX-6p-2rbs-Aster-C$_{318-504}$		Recombinant expression
pET22b-His6-STARD1$_{66-284}$		Recombinant expression
**sgRNA**	**Sequence (5’ to 3’)**	**Application**
AAVS1_1	GCTCCGGAAAGAGCATCCT	CRISPR screen validation, dropout screen
AAVS1_2	GCTGTGCCCCGATGCACAC	CRISPR screen validation, dropout screen
ARMH3_1	GCACATACTAAAGGACGGCA	CRISPR screen validation
ARMH3_2	GATGAACACAGGCTTCACAG	CRISPR screen validation
ARMH3_3	CACTTACGGTCACTAAGCAA	CRISPR screen validation
PITPNB_1	GCTTACTTGTTCTACAGTAG	CRISPR screen validation
PITPNB_2	ATGGTTTAGATCCAAACACA	CRISPR screen validation
PITPNB_3	TGTCAATCCAACAAAAAAGC	CRISPR screen validation
OSBP_1	GAAATGAGACATACCTGCCG	Dropout screen
OSBP_2	GTTCCTTTACCTGTTCACAG	Dropout screen
OSBP2_1	AGTGGAGCAGATGTGCCTGG	Dropout screen
OSBP2_2	AGCCTCTTCTCAAACCACAG	Dropout screen

Supplementary Table 8. Primers used for PCR-amplification of sgRNAs in the genome-wide CRISPR/Cas9 screen.

Primer	Sequence (5' to 3')	Application
Brunello_P5_stag0	AATGATACGGCGACCACCGAGATCTACACTCTTTCCCTACACGACGCTCTTCCGATCTTTGTGGAAAGGACGAAACACCG	P5 primer mix
Brunello_P5_stag1	AATGATACGGCGACCACCGAGATCTACACTCTTTCCCTACACGACGCTCTTCCGATCTCTTGTGGAAAGGACGAAACACCG	P5 primer mix
Brunello_P5_stag2	AATGATACGGCGACCACCGAGATCTACACTCTTTCCCTACACGACGCTCTTCCGATCTGCTTGTGGAAAGGACGAAACACCG	P5 primer mix
Brunello_P5_stag3	AATGATACGGCGACCACCGAGATCTACACTCTTTCCCTACACGACGCTCTTCCGATCTAGCTTGTGGAAAGGACGAAACACCG	P5 primer mix
Brunello_P5_stag4	AATGATACGGCGACCACCGAGATCTACACTCTTTCCCTACACGACGCTCTTCCGATCTCAACTTGTGGAAAGGACGAAACACCG	P5 primer mix
Brunello_P5_stag6	AATGATACGGCGACCACCGAGATCTACACTCTTTCCCTACACGACGCTCTTCCGATCTTGCACCTTGTGGAAAGGACGAAACACCG	P5 primer mix
Brunello_P5_stag7	AATGATACGGCGACCACCGAGATCTACACTCTTTCCCTACACGACGCTCTTCCGATCTACGCAACTTGTGGAAAGGACGAAACACCG	P5 primer mix
Brunello_P5_stag8	AATGATACGGCGACCACCGAGATCTACACTCTTTCCCTACACGACGCTCTTCCGATCTGAAGACCCTTGTGGAAAGGACGAAACACCG	P5 primer mix
Brunello_P7_N701	CAAGCAGAAGACGGCATACGAGAT TCGCCTTAGTGACTGGAGTTCAGACGTGTGCTCTTCCGATCTTCTACTATTCTTTCCCCTGCACTGT	Barcode for DMSO-treated samples
Brunello_P7_N706	CAAGCAGAAGACGGCATACGAGAT CATGCCTAGTGACTGGAGTTCAGACGTGTGCTCTTCCGATCTTCTACTATTCTTTCCCCTGCACTGT	Barcode for orpinolide-treated samples

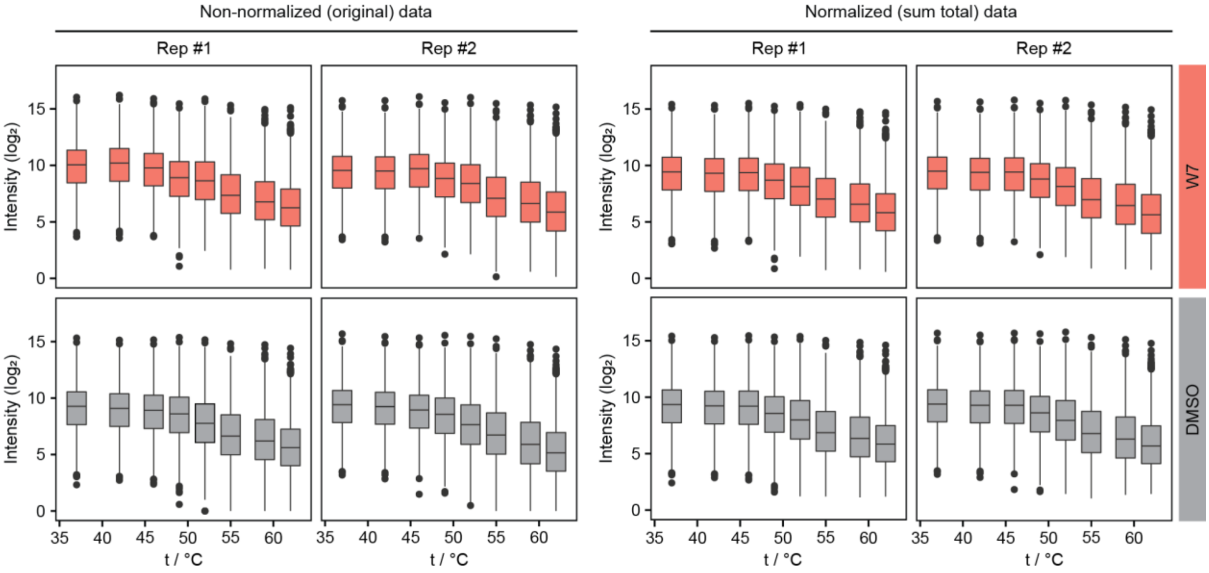

Supplementary Figure 1. Thermal proteome profiling. Non-normalized (left) and sum total normalized (right) distribution of the TPP data for individual W7/DMSO-treated biological replicates (n = 2). The solid line in the box plots represents the median, box limits show interquartile range (IQR) and its whiskers 1.5 x IQR. Outliers are represented as block dots. See also Figure 3, Extended Data Fig. 6 and Supplementary Table 5 for further information.

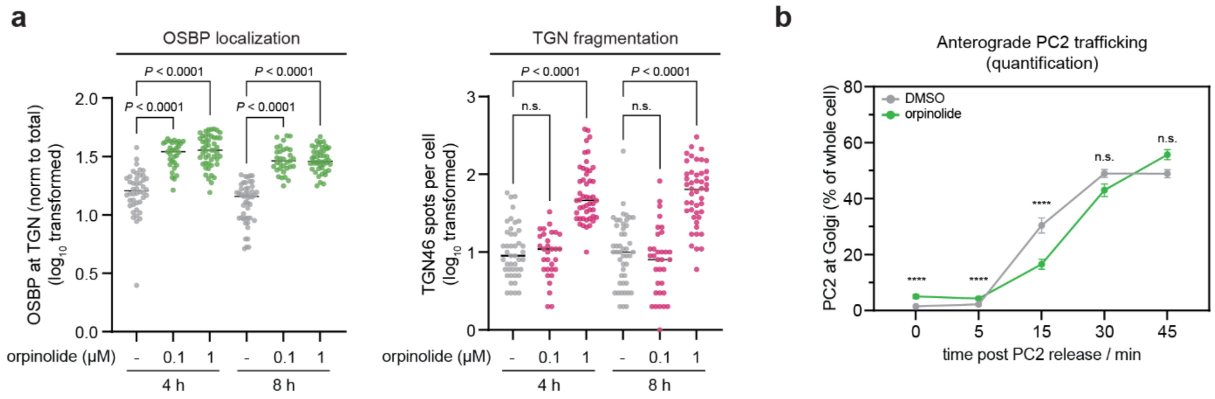

Supplementary Figure 2. Quantification of Golgi morphology alterations upon orpinolide treatment. (a) Quantification of OSBP localization to the Golgi as identified by TGN46 (left) and *trans*-Golgi fragmentation measured by the number of TGN46 spots per cell (right). The graphs present the log10 transformed data depicted in Extended Data Fig. 8, which was utilized for the determination of statistical significance. One-way ANOVA was performed on log10 transformed data; $P < 0.0001$ with Dunnett's multiple comparisons test (annotated on graphs). **(b)** Quantification of PC2 localization to the Golgi, normalized to total PC2 per cell. Graph shows mean ± s.e.m. data from 3 independent experiments, with between 42-58 cells per condition. Two-way ANOVA was performed on log10 transformed data (depicted in Figure 5a) with P (Interaction) < 0.0001 and P (Treatment factor) < 0.01. Šídák's multiple comparison test compares DMSO against orpinolide at each timepoint with **** representing $P < 0.0001$; n.s. $P = 0.8432$ (4h); n.s. $P = 0.7773$ (8h).

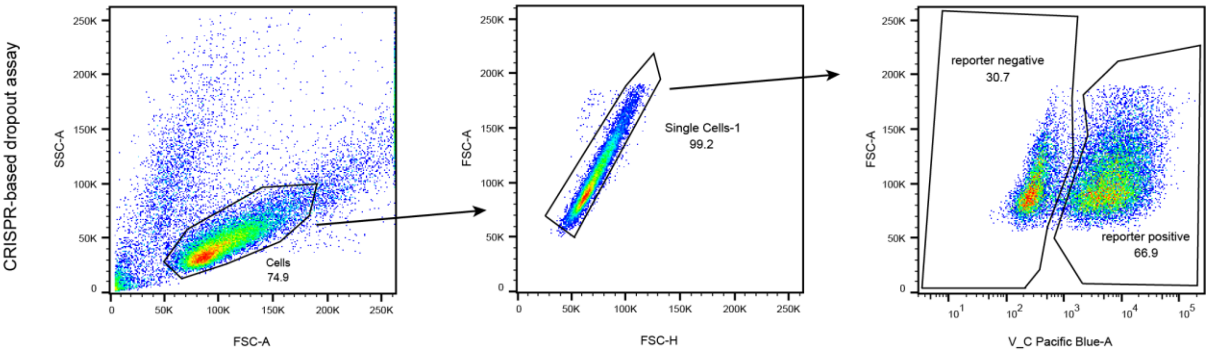

Supplementary Figure 3. Gating strategy used for flow cytometry experiments. Gating strategy used for the CRISPR-based dropout experiment shown in Figure 4f and Extended Data Fig. 9d.

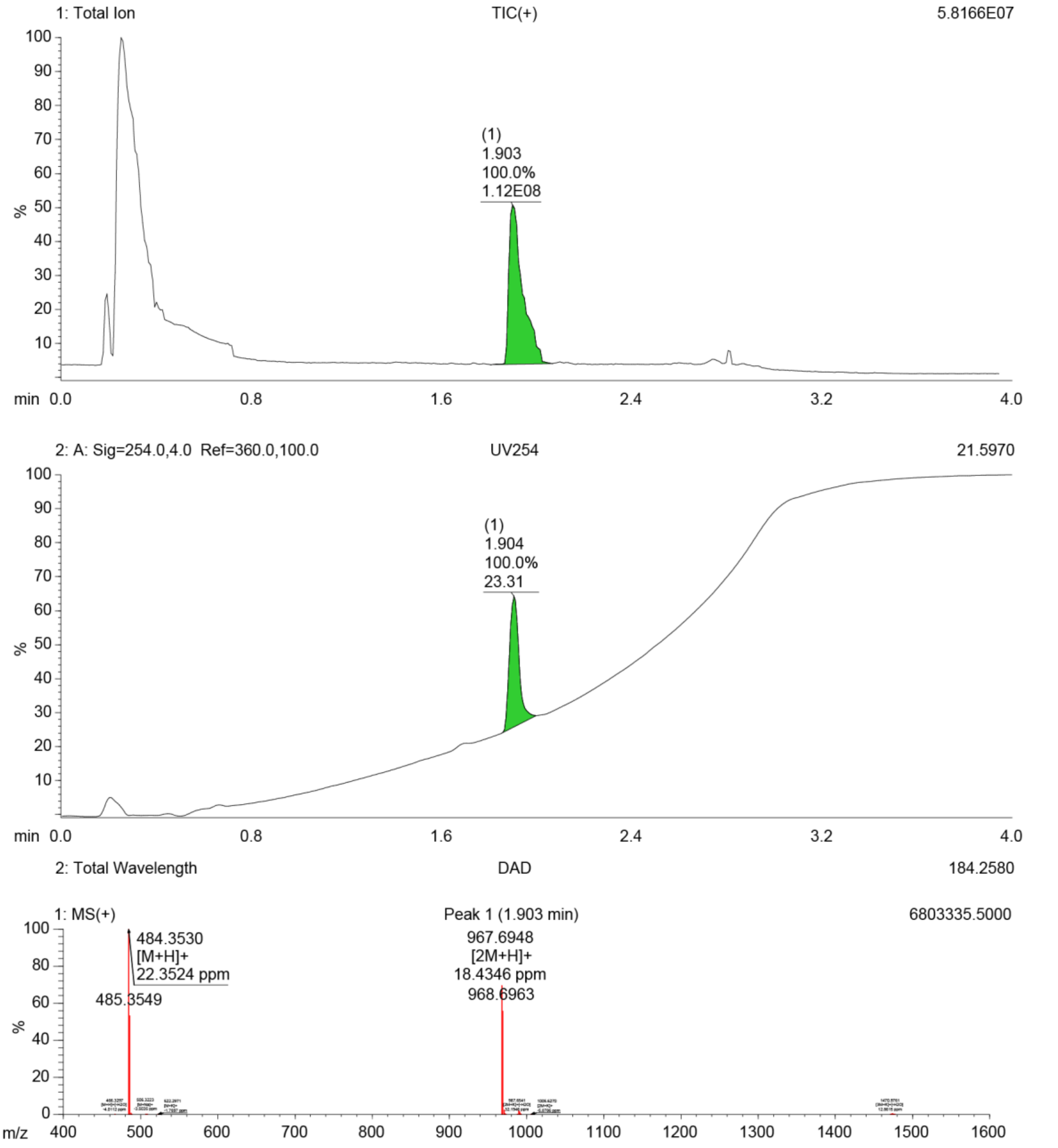

Peak #	Chrom	Time	Mass Found	State	Area %BP	TIC	BPI	BPM	Error PPM	Error mDa
1	1:MS…	1.903	483.3349		0.0	1.96E07	6803335.50	484.3530	-6.4287	-9.4695

Supplementary Figure 4. Purity of orpinolide (W7) analyzed via LC-MS.
