# Supplementary material for: Orpinolide disrupts a leukemic dependency on cholesterol transport by inhibiting OSBP
Source: Nat Chem Biol. 2024 Jun 21;21(2):193–202. doi: 10.1038/s41589-024-01614-4 (PMC11782089; doi:10.1038/s41589-024-01614-4)
Supplement: Supplementary file 2 — Reporting Summary [file 41589_2024_1614_MOESM2_ESM.pdf]

nature portfolio

Corresponding author(s): Georg E. Winter, Herbert Waldmann

Last updated by author(s): Mar 30, 2024

Reporting Summary

Nature Portfolio wishes to improve the reproducibility of the work that we publish. This form provides structure for consistency and transparency in reporting. For further information on Nature Portfolio policies, see our Editorial Policies and the Editorial Policy Checklist.

Statistics

For all statistical analyses, confirm that the following items are present in the figure legend, table legend, main text, or Methods section.

n/a	Confirmed	
☐	☒	The exact sample size (*n*) for each experimental group/condition, given as a discrete number and unit of measurement
☐	☒	A statement on whether measurements were taken from distinct samples or whether the same sample was measured repeatedly
☐	☒	The statistical test(s) used AND whether they are one- or two-sided<br>*Only common tests should be described solely by name; describe more complex techniques in the Methods section.*
☒	☐	A description of all covariates tested
☒	☐	A description of any assumptions or corrections, such as tests of normality and adjustment for multiple comparisons
☐	☒	A full description of the statistical parameters including central tendency (e.g. means) or other basic estimates (e.g. regression coefficient) AND variation (e.g. standard deviation) or associated estimates of uncertainty (e.g. confidence intervals)
☐	☒	For null hypothesis testing, the test statistic (e.g. *F*, *t*, *r*) with confidence intervals, effect sizes, degrees of freedom and *P* value noted<br>*Give P values as exact values whenever suitable.*
☒	☐	For Bayesian analysis, information on the choice of priors and Markov chain Monte Carlo settings
☒	☐	For hierarchical and complex designs, identification of the appropriate level for tests and full reporting of outcomes
☒	☐	Estimates of effect sizes (e.g. Cohen's *d*, Pearson's *r*), indicating how they were calculated

Our web collection on statistics for biologists contains articles on many of the points above.

Software and code

Policy information about availability of computer code

Data collection	Cell viability and HiBiT-CETSA assays: Multilabel Plate Reader Platform Victor X3 model 2030 (PerkinElmer).<br>Fluorescence measurements: Spark Cyto multimode microplate reader (Tecan).<br>Mass spectrometry: Orbitrap Fusion Lumos Tribrid mass spectrometer coupled to a Dionex Ultimate 3000 RSLCnano system and operated via Xcalibur (v3.3.2782.34 or v4.3.73.11) and Tune (v3.3 or v3.4.3072.18).<br>NGS: Illumina HiSeq4000 (https://www.illumina.com/).<br>Confocal microscopy: SP5 scanning confocal (Leica) with 63x/1.4 Plan Apo oil objective lens (Zeiss), with pixel size of 0.161 µm and step size 0.4 µm.<br>Flow cytometry: Data was collected on a LSRFortessa (BD Biosciences) using BD FACSDiva software (v9.0).

Data analysis	GraphPad Prism (v9.5.1) was used for statistical analyses.<br>Expression proteomics: Proteome Discoverer (v2.4.1.15) was used for processing of acquired data. Searches were performed with full tryptic digestion against the human SwissProt database (Homo sapiens (SwissProt TaxID=9606) (v2017), 42252 sequences).<br>Thermal proteome profiling: Nonparametric analysis of response curves (NPARC) R-package (v1.2.0; Childs et al, Mol Cell Proteomics, 2019) was used to identify significant shifts in protein melting behavior upon compound treatment.<br>Genome-wide CRISPR/Cas9 screens and RNA-seq: BamTools (v2.5.2), Cutadapt (v3.4), fastx toolkit (v0.0.14), Bowtie2 (v2.4.4), STARS algorithm (v1.3), Trimmomatic (v0.32), STAR aligner (v2.5.2b), htseq-count command (v0.11.2), DESeq2 (v1.34.0). The data analysis codes are available at https://github.com/GWinterLab/W7.<br>Protein interaction analysis: Data analysis was conducted with the statistical software R (version R-4.2.0) while the network was visualized using the software Cytoscape (v.3.8.0).<br>Microscopy: Fiji ImageJ2 (National Institute of Health).<br>Flow cytometry analysis: FlowJo (v10.8.1).

For manuscripts utilizing custom algorithms or software that are central to the research but not yet described in published literature, software must be made available to editors and reviewers. We strongly encourage code deposition in a community repository (e.g. GitHub). See the Nature Portfolio guidelines for submitting code & software for further information.

Data

Policy information about availability of data

All manuscripts must include a data availability statement. This statement should provide the following information, where applicable:

- Accession codes, unique identifiers, or web links for publicly available datasets
- A description of any restrictions on data availability
- For clinical datasets or third party data, please ensure that the statement adheres to our policy

The mass spectrometry proteomics data (Figures 2a-2b and 4a, Extended Data Fig. 4 and 6, Supplementary Tables 2 and 5) have been deposited to the ProteomeXchange Consortium via the PRIDE80 partner repository with the dataset identifier PXD040694 and 10.6019/PXD040694 (expression proteomics) as well as PXD040692 and 10.6019/PXD040692 (thermal proteome profiling). Raw and analyzed RNA-seq and genome-wide CRISPR/Cas9 screening datasets (Figures 2c-2d and 3, Extended Data Fig. 5, Supplementary Tables 3 and 4) are available in NCBI's Gene Expression Omnibus under accession number GSE226849. Additionally, publicly available data from the following databases was used in this study as well: DepMap (22Q4 and 23Q2), The Human Protein Atlas project (v22.0), UniProtKB (13.01.2023) and BioGRID (v4.4.212). The data supporting all of the findings in this study are available within the paper, its supplementary files, and the mentioned databases.

Human research participants

Policy information about studies involving human research participants and Sex and Gender in Research.

Reporting on sex and gender	N/A
Population characteristics	N/A
Recruitment	N/A
Ethics oversight	N/A

Note that full information on the approval of the study protocol must also be provided in the manuscript.

Field-specific reporting

Please select the one below that is the best fit for your research. If you are not sure, read the appropriate sections before making your selection.

☒ Life sciences ☐ Behavioural & social sciences ☐ Ecological, evolutionary & environmental sciences

For a reference copy of the document with all sections, see nature.com/documents/nr-reporting-summary-flat.pdf

Life sciences study design

All studies must disclose on these points even when the disclosure is negative.

Sample size	All presented data is based on cultured human cell lines. Sample sizes were not predetermined using statistical analyses. Sample sizes were based on prior experience in the field and our previous studies (Mayor-Ruiz et al, Mol Cell, 2019; Mayor-Ruiz et al, Nat Chem Biol, 2020).
Data exclusions	In quantitative proteomics, only proteins with > 1 peptide and > 1 protein unique peptide detected were considered for downstream analysis. In thermal proteome profiling, only proteins quantified ≥ 2 PSMs across the vehicle (DMSO) and compound treated conditions were considered.
Replication	Unless stated in figure legends or method sections, all experiments were done at least twice to ensure reproducibility. The number of

Replication	technical replicates and/or independent biological experiments are specified in the respective figure legends.
Randomization	In general, all samples were numerically labeled and randomized before compound treatments. Post-treatment processing of samples occured in one batch in random orders. All experiments were carried out with appropriate internal negative and/or positive controls. Most results were backed up with orthogonal methods to have confidence in the validity of obtained results (as described in the manuscript).
Blinding	No blinding was performed as no subjective measurements were done.

Reporting for specific materials, systems and methods

We require information from authors about some types of materials, experimental systems and methods used in many studies. Here, indicate whether each material, system or method listed is relevant to your study. If you are not sure if a list item applies to your research, read the appropriate section before selecting a response.

Materials & experimental systems

n/a	Involved in the study
☐	☒ Antibodies
☐	☒ Eukaryotic cell lines
☒	☐ Palaeontology and archaeology
☒	☐ Animals and other organisms
☒	☐ Clinical data
☒	☐ Dual use research of concern

Methods

n/a	Involved in the study
☒	☐ ChIP-seq
☐	☒ Flow cytometry
☒	☐ MRI-based neuroimaging

Antibodies

Antibodies used	All antibodies were purchased from the sources cited in the manuscript.<br>Western blotting:<br>The following primary antibodies were used: OSBP (1:2,000; Bethyl, A304-553A), GAPDH (1:5,000; Santa Cruz Biotechnology, sc-365062), GOLIM4 (1:1,000; Thermo Scientific, PA5-51624), PITPNB (1:2,000; Bethyl, A305-591A), GALNT2 (1:1,000; Abcam, ab262868), p-Akt (1:2,000; Cell Signaling Technology, 4060T), Akt (1:1,000; Cell Signaling Technology, 4691T), HA (1:5,000; Cell Signaling Technology, 3724S). Secondary antibody: Peroxidase-conjugated AffiniPure Goat Anti-Rabbit IgG (1:10,000; Jackson ImmunoResearch 111-035-003).<br>Immunofluorescence microscopy:<br>The following primary antibodies were used: OSBP (1:150; Atlas Antibodies, HOA039227), TGN46 (1:200; Bio-Rad, AHP500GT), PC2 (recognizes folded PC2 only; 1:200; Hybridoma Bank, II6B3), Giantin (1:100; Institut Curie, recombinant proteins platform, A-R-R#05). Secondary antibodies: Alexa Fluor® 488-, 568- and 647-conjugated secondary antibodies (Invitrogen, goat anti-rabbit-488 A11008, goat anti-mouse-488 A11001, goat anti-mouse-568 A11031, donkey anti-sheep-647 A21448). 1:400 dilutions.
Validation	Western blotting:<br>Target specificity for OSBP antibody was validated in CETSA experiments (Figure 4c and Extended Data Fig. 7). GOLIM4, GALNT2, p-Akt and Akt antibodies was validated by the expected protein level changes upon treatment of cells with W7 (Extended Data Fig. 4d, Extended Data Fig. 7c, Extended Data Fig. 10b-10d). PITPNB antibody was validated in CRISPR-induced sgPITPNB KBM7 cell pools (Extended Data Fig. 5c). Validations as well as hundreds of references for GAPDH, HA and Peroxidase-conjugated AffiniPure Goat Anti-Rabbit IgG can be found in the vendor sites using above specified catalogue numbers.<br>Immunofluorescence microscopy:<br>Target specificity for OSBP and PC2 antibodies were validated in Golgi fragmentation or anterograde trafficking assays (Figure 5a-5b, Extended Data Fig. 8a-8d). Validations as well as references for TGN46, Giantin as well as Alexa Fluor® 488-, 568- and 647-conjugated secondary antibodies can be found in the vendor sites using above specified catalogue numbers.

Eukaryotic cell lines

Policy information about cell lines and Sex and Gender in Research

Cell line source(s)	Cell line culture conditions are stated in the Methods section of the manuscript. KBM7 cells (from T. Brummelkamp lab (Carette et al, Science, 2009). MOLT4, Jurkat, LOUCY and P-12 Ichikawa cells (from James E. Bradner lab (Winter et al, Mol Cell, 2017). 293T, 768-O, A375, A673, AsPC1, BxPC3, DU145, H1299, HCT116, HeLa, HT29, K562, LNCaP, MCF7, MiaPaCa2, MV4;11, NALM6, NCI-H358, OCIAML3, Rh30, RKO, RPE-1, SK-E-S1, SK-N-SH and U2OS cells were obtained from ATCC. KBM7 and Jurkat cells constitutively expressing Cas9 (from Mayor-Ruiz et al, Nat Chem Biol, 2020). RCS cells were a gift from Carmine Settembre (TIGEM; King & Kimura, J Cell Biochem, 2003). KBM7 iCas9 cells were a gift from Johannes Zuber (IMP - Research Institute of Molecular Pathology). Non-malignant PBMCs were isolated from peripheral blood of a healthy adult volunteer (purchased from the local transfusion service, Red Cross Austria).
Authentication	All used cell lines were authenticated by vendors and routinely authenticated via cell morphology. MV4;11 cells were further authenticated via short tandem repeat profiling.
Mycoplasma contamination	All used cell lines were routinely tested and confirmed negative for mycoplasma contamination.

Commonly misidentified lines (See ICLAC register)	No commonly misidentified cell lines were used.

Flow Cytometry

Plots

Confirm that:

☒ The axis labels state the marker and fluorochrome used (e.g. CD4-FITC).

☒ The axis scales are clearly visible. Include numbers along axes only for bottom left plot of group (a 'group' is an analysis of identical markers).

☒ All plots are contour plots with outliers or pseudocolor plots.

☒ A numerical value for number of cells or percentage (with statistics) is provided.

Methodology

Sample preparation	Cultured human cancer cell lines were used. To quantify the influence of OSBP and/or ORP4 genetic perturbation, KBM7 cells expressing inducible Cas9 (iCas9) or Jurkat cells constitutively expressing Cas9 were transduced with pLenti-U6-sgRNA#1-U6-sgRNA#2-EF1αs-eBFP2 reporter with 50-60% infection efficiency. The reporter plasmid carries a combination of sgRNAs targeting OSBP, OSBP2 or AAVS1 locus to yield single or dual OSBP/OSBP2 knockouts (see Supplementary Table 7). In KBM7 iCas9 cells, infection levels were determined by flow cytometry three days after transduction based on BFP marker expression (Day 0) and Cas9 expression was induced with doxycycline (0.4 µg/mL). In Jurkat-Cas9 cells, infection levels were determined by flow cytometry three days after transduction. In both cases, the percentage of sgRNA+ (BFP+) cells were monitored by flow cytometry in regular intervals. Flow cytometry measurements were performed on LSRFortessa (BD Biosciences) while the data was analyzed in FlowJo (v10.8.1). For flow cytometry gating strategies see Supplementary Figure 3.
Instrument	All flow cytometric analyses were performed on BD LSRFortessa (4 laser, 16 detector configuration; BD Bioscience).
Software	BD FACSDiva software (v9.0), FlowJo (v10.8.1)
Cell population abundance	Cultured human cancer cell lines were used.
Gating strategy	Forward scatter area vs. side scatter area plot was used to separate cell events from debris and dead cells. Forward scatter height vs. forward scatter area were used to separate single cells from aggregates. V_C-Pacific Blue-A was used to distinguish BFP+ and BFP- cells. An example of gating strategy used for all FACS experiments is provided in Supplementary Figure 3.

☒ Tick this box to confirm that a figure exemplifying the gating strategy is provided in the Supplementary Information.
