# Supplementary material for: Orpinolide disrupts a leukemic dependency on cholesterol transport by inhibiting OSBP
Source: Nat Chem Biol. 2024 Jun 21;21(2):193–202. doi: 10.1038/s41589-024-01614-4 (PMC11782089; doi:10.1038/s41589-024-01614-4)

Source Data Ext. Data Fig. 5c. Uncropped western blots.

Molecular weight standard: Color Prestained Protein Standard, broad range (10-250 kDa; NEB, P7719)

5c

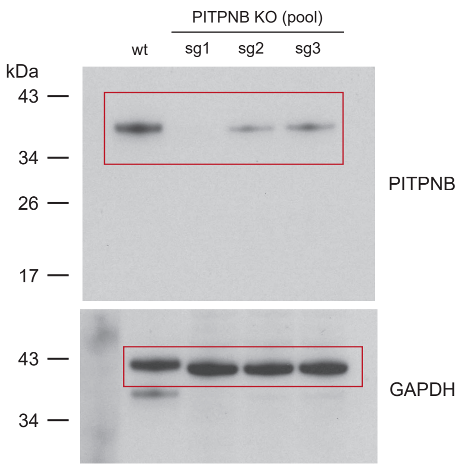

Supplement: Supplementary file 22 — Unprocessed western blots. [file 41589_2024_1614_MOESM22_ESM.pdf]
