# Supplementary material for: Orpinolide disrupts a leukemic dependency on cholesterol transport by inhibiting OSBP
Source: Nat Chem Biol. 2024 Jun 21;21(2):193–202. doi: 10.1038/s41589-024-01614-4 (PMC11782089; doi:10.1038/s41589-024-01614-4)

Source Data Ext. Data Fig. 7. Uncropped western blots for Ext. Data Figs. 7a, 7c and 7g.

Molecular weight standard: Color Prestained Protein Standard, broad range (10-250 kDa; NEB, P7719)

7a

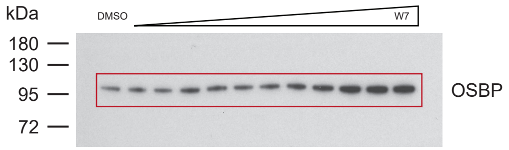

7c

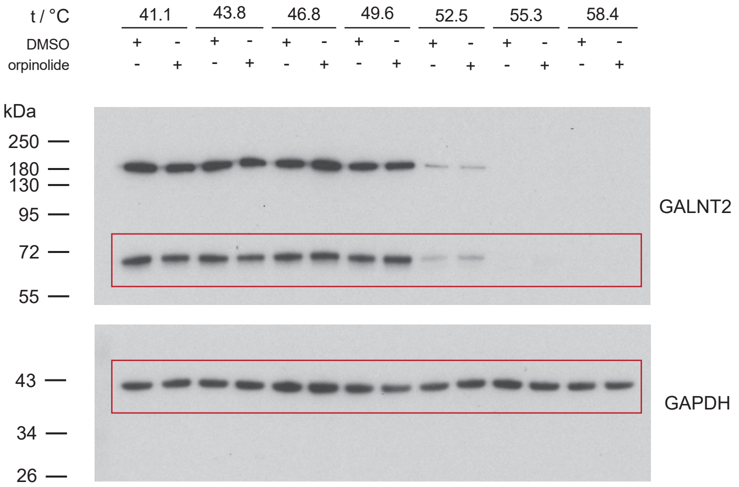

7g

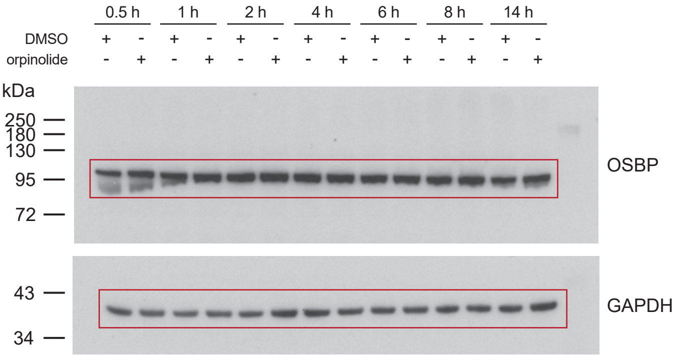

Supplement: Supplementary file 25 — Unprocessed western blots. [file 41589_2024_1614_MOESM25_ESM.pdf]
