# Supplementary material for: Orpinolide disrupts a leukemic dependency on cholesterol transport by inhibiting OSBP
Source: Nat Chem Biol. 2024 Jun 21;21(2):193–202. doi: 10.1038/s41589-024-01614-4 (PMC11782089; doi:10.1038/s41589-024-01614-4)

Source Data Extended Data Fig. 10. Uncropped western blots for Ext. Data Fig. 10b-10c.

Molecular weight standard: Color Prestained Protein Standard, broad range (10-250 kDa; NEB, P7719)

10b

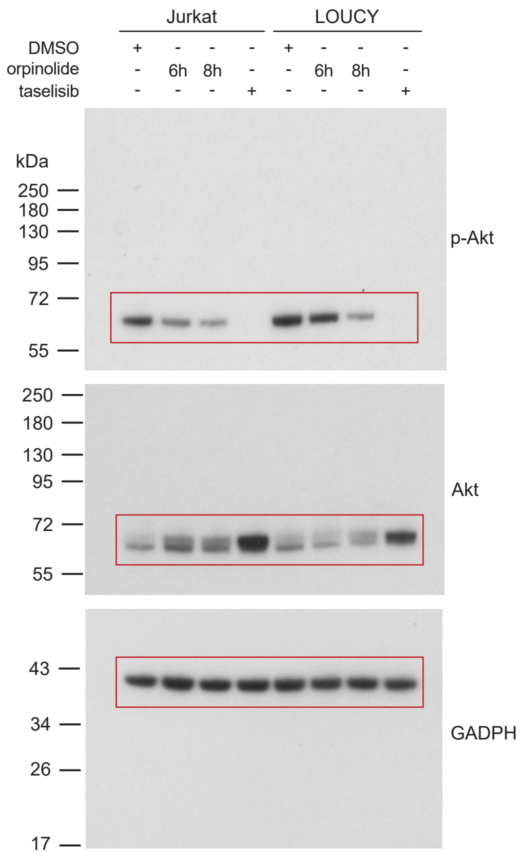

10c

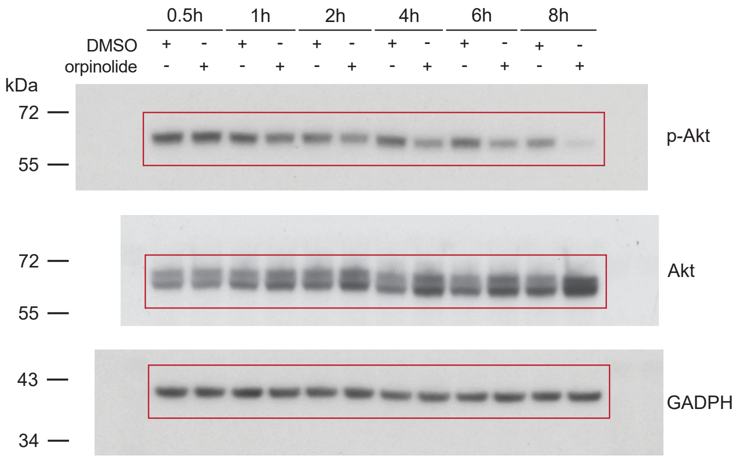

Supplement: Supplementary file 29 — Unprocessed western blots. [file 41589_2024_1614_MOESM29_ESM.pdf]
